# Supplementary material for: Critical length screening enables 19% efficiency in thick-film organic solar cells
Source: Nat Commun. 2025 Nov 7;16:9840. doi: 10.1038/s41467-025-64808-x (PMC12594902; doi:10.1038/s41467-025-64808-x)
Supplement: Supplementary file 1 — Supplementary Information [file 41467_2025_64808_MOESM1_ESM.pdf]

Supplementary Information

Critical Length Screening Enables 19% Efficiency in Thick-Film Organic Solar Cells

Yuan Meng[1], Bo Cheng[2], Dongcheng Jiang[1], Jiangkai Sun[1], Jiawei Qiao[1], Beibei Shi[1], Haisheng Ma[3], Jingtian Zhu[1], Lianbo Wang[1], Runzheng Gu[1], Peng Lu[1], Yanna Sun[4]*, Xiaoyan Du[1], Xia Guo[2], Ke Gao[4], He Yan[5], Maojie Zhang[2]*, Feng Chen[1], Yanming Sun[3], Xiaotao Hao[1]*, Hang Yin[1]*

Affiliations:

[1] School of Physics, State Key Laboratory of Crystal Materials, Shandong University, Jinan, Shandong, 250100 China

[2] School of Chemistry & Chemical Engineering, National Engineering Research Center for Colloidal Materials, Shandong University, Jinan, Shandong, 250100 China

[3] School of Chemistry and Environment, Heeger Beijing Research and Development Center, Beihang University, Beijing, 100191 China

[4] School of Chemistry and Chemical Engineering, Shandong Provincial Key Laboratory for Science of Material Creation and Energy Conversion, Science Center for Material Creation and Energy Conversion, Institute of Frontier Chemistry, Shandong University, Qingdao, Shandong, 266237 China

[5] Department of Chemistry, Hong Kong University of Science and Technology, Clear Water Bay, Kowloon, Hong Kong, 999077 China

*Corresponding authors. Emails: ynsun@sdu.edu.cn (Yanna Sun); mjzhang@sdu.edu.cn (Maojie Zhang); haoxt@sdu.edu.cn (Xiaotao Hao); hyin@sdu.edu.cn (Hang Yin).

Keywords: Critical length, Screening methodology, Thick-film organic solar cells.

This PDF file includes:

Supplementary Methods

Supplementary Figures 1-31

Supplementary Tables 1-19

Supplementary References.

Supplementary Methods

Extracting acceptor domain size from GISAXS images

1D GISAXS profiles are extracted on the basis of Debye-Anderson Brumberger (DAB) and fractal models using the fitting software SASView (Version 4.2.2). The first term of DAB is used to simulate the scattering from the polymer domain, where ξ represents the average correlation length, q is the scattering wave vector, and A_1 is a fitting parameter determined independently. The second term corresponds to the fractal model, which describes the occupation of a fractal-like structure in the non-fullerene acceptor. P(q, R) and S(q, R) are the form factor and fractal structure factor, respectively. The correlation length and fractal dimension of the fractal-like acceptor aggregates are denoted by η and D, respectively. The Guinier radius (R_g) is used to characterize the average domain size of the acceptor phase.

$$\mathrm{I}(q) = \frac{A_1}{[1+(q\xi)^2]^2} + A_2\langle P(q,R)\rangle S(q,R,\eta,D) + B \tag{1}$$

$$\mathrm{S}(q) = 1 + \frac{\sin[(D-1)\tan^{-1}(q\eta)]b\pm\sqrt{b^2-4ac}}{(qR)^D}\frac{D\Gamma(D-1)}{[1+\frac{1}{q\eta^2}]^{\frac{(D-1)}{2}}} \tag{2}$$

$$R_g = \sqrt{(\frac{D(D+1)}{2})}\eta \tag{3}$$

Film-depth-dependent light absorption spectroscopy (FLAS) data processing

The law of Beer-Lambert is:

$$A = -\log(\frac{I_T}{I_0 - I_R}) \tag{4}$$

where A is the optical density, I_0 is the incident light density, I_R is the reflected light intensity and I_T is the transmitted light intensity. I_T can be obtained via:

$$I_T = (I_0 - I_R) \times 10^{-A} \tag{5}$$

By controlling the etching time, the active layer can be selectively etched from each sub-layer using soft plasma generated by oxygen glow discharge. The etching process is continuously monitored in situ with a light absorption spectrometer. Assuming the absorbance of each sub-layer is A_1, A_2, ..., A_n, the total transmitted light intensity can be calculated by:

$$I_T = (I_0 - I_R) \prod_{i=1}^{n} 10^{-A_i} = (I_0 - I_R) \times 10^{-\sum_{i=1}^{n} A_i} \tag{6}$$

Then, we can get:

$$A = A_1 + A_2 + A_3 + \cdots + A_n \tag{7}$$

The absorbance of the whole active layer is the sum of absorbance of all sublayers. Consequently, we can get the absorption spectra of all the sub-layers at different depths of active layer.

The calculation of trap density (N_t)

We consider only materials with a depleted active layer and a frequency-independent capacitance within our frequency range as the dielectric constant.[1] The dielectric constant is evaluated based on the material's geometric capacitance, which reflects the capacitance measured when the contribution is solely from the material itself, including electronic, atomic, and ionic polarization. If the capacitance exhibits minimal frequency dependence, the geometric capacitance is measured at 10^6 Hz. Once the geometric capacitance is determined, the relative permittivity is calculated using the film's

thickness L and device area A:

$$\varepsilon = \frac{C_g L}{\varepsilon_0 A} \tag{8}$$

At low frequencies, the capacitance resulting from trapping and de-trapping processes provides information about the lower limit of the trap density. Trap states can only be probed at low frequencies (around 10 kHz). Assuming that the additional capacitance near the built-in voltage (V_{bi}) is due to trapped carriers being released to the mobility edges, the trapped-charge density (N_{CV}) can be calculated by:

$$N_{CV} = -\frac{C^3}{\varepsilon q A^2}\left(\frac{dC}{dV}\right)^{-1} \tag{9}$$

Then, the trapped charge density of the accessible trap state can be estimated by:

$$\frac{1}{C^2} \propto -\frac{2}{N_t \varepsilon q A^2} V \tag{10}$$

Poole-Frenkel model

The Poole-Frenkel model describes the behavior of trapped carriers that are released under the influence of a strong electric field. This strong electric field can reduce the effective depth of the trap, enabling carriers to escape. The relationship between mobility and the electric field is expressed by:

$$\mu(F) = \mu_0 \exp(0.89\beta\sqrt{F}) \tag{11}$$

where μ_0 implies the zero-field mobility, β is the Poole-Frenkel slope, represents the field-dependent of mobility.

The Poole-Frenkel (PF) model[2] can be expressed as:

$$J = \frac{9}{8}\varepsilon_r \varepsilon_0 \frac{V^2}{L^3}\mu_0 \exp(0.89\beta\sqrt{F}) \tag{12}$$

where ε_r is the relative permittivity, ε_0 is the vacuum permittivity, L is the thickness of active layer, μ_0 is the zero-field mobility, β is the field-dependent of mobility and F is the electric field intensity.

Critical Length Description:

Our analysis employed the alternating current (AC) conductivity framework, revealing

two distinct conduction regimes. In the low-frequency domain, conductivity remains constant and corresponds to the direct current (DC) conductivity. However, as the frequency of the applied alternating voltage increases progressively, the conductivity exhibits an exponential dependence on frequency, a characteristic behavior of the AC conductivity region. The universal response of AC conductivity emerges from the combined effects of static and dynamic disorder; however, their dominance varies across different frequency ranges and temperatures. In the low-frequency region, charge carrier hopping conduction is primarily governed by static disorder due to localized states, while energy fluctuations caused by dynamic disorder may assist carriers in overcoming static disorder barriers, influencing the high-frequency response through energy fluctuations and polarization effects.

The concept of critical length L_C was originally proposed by Papathanassiou et al. to describe the distribution of conductive pathways in the universal response model of AC conductivity. It is defined as the ratio of the average carrier velocity v to the hopping frequency ω_H (or the inverse of the hopping time):

$$L_C = \frac{v}{\omega_H} \tag{13}$$

Carrier transport within the active layer is governed on one hand by the intrinsic mobility of carriers under zero field conditions, and on the other hand by phenomena such as defect states and Coulomb potential fluctuations caused by static charges within the film. These effects result in an overall fluctuating energy landscape that impacts carrier migration and diffusion pathways.

The influence of defect states on carrier transport speed manifests in the magnitude of the mobility field dependence, represented by the filed-dependent of mobility β in the Poole-Frenkel model. In the derivation of the Poole-Frenkel model, β quantifies the

extent to which an applied electric field lowers the potential barrier of an isolated defect state, which is related to the dielectric properties of the material. Under weak electric fields, a large filed-dependent of mobility (high β) corresponds to lower carrier mobility, which is unfavorable for long-range carrier transport. From a macroscopic perspective, β reflects the degree of fluctuation in the energy landscape: a higher β value corresponds to the stronger field-dependent mobility and greater fluctuations in the energy landscape, indicating increased sensitivity of carrier transport to the energy environment.[3]

Considering that β characterizes both the carrier escape capability from individual defect states and the influence of the global energy landscape on carrier transport pathways, we incorporate β squared in our model. By combining μ_0 and β, we construct a characteristic velocity of charge carriers. Dimensional analysis further confirms that this characteristic velocity is consistent with the average carrier velocity. This formulation better captures the carrier transport behavior in organic photovoltaic devices under non-steady-state electric fields and complex energy landscapes.

Experimentally, we calculated the critical lengths for various acceptors ranging from fullerene to A-D-A type and Y-series materials. A strong correlation between critical length and thick-film device performance was observed. Using the critical length as a screening parameter, we fabricated a series of high-performance thick-film devices, thereby validating the effectiveness of this criterion.

Moreover, in AC conductivity measurements, the applied voltage is sinusoidal, alternating between forward and reverse field directions. These opposing field components alternately facilitate and hinder carrier extraction at the cathode. To account for this in the estimation of critical length, only the positive half-cycle of the sinusoidal field is considered, which results in adopting a half-period in the

denominator (t = 1/2f = 1/2ω). Consequently, the complete expression for critical length includes 2ω_H.

In summary, the critical length of charge carriers in organic solar cells is calculated using Equation (1) presented in the manuscript.

Calculation of the Relative Change in Hopping Frequency (Δ):

The relative change Δ is defined as the variation in hopping frequency when the applied voltage increases from 1 V to 2 V. We have included the formula for Δ in the supplementary information.

Δ is calculated as the difference between the hopping frequencies at these two voltages divided by the hopping frequency at 1 V, representing the normalized, dimensionless change relative to the hopping frequency at 1 V. The calculation formula is as follows:

$$\Delta = \frac{\omega_{H2} - \omega_{H1}}{\omega_{H1}} \tag{14}$$

ω_{H2} denotes the hopping frequency of charge carriers under an applied voltage of 2 V, while ω_{H1} corresponds to that under 1 V.

Supplementary Figures

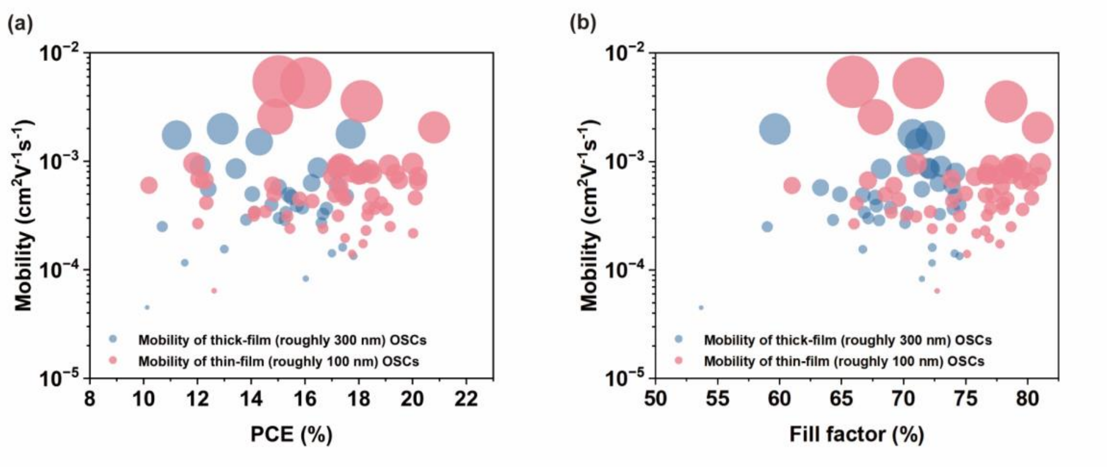

Supplementary Fig. 1. Comparison of power conversion efficiency (PCE), fill factor (FF) and electron mobility between thick-film organic solar cells and thin-film organic solar cells.[4–93] (a) PCE and electron mobility. (b) FF and electron mobility.

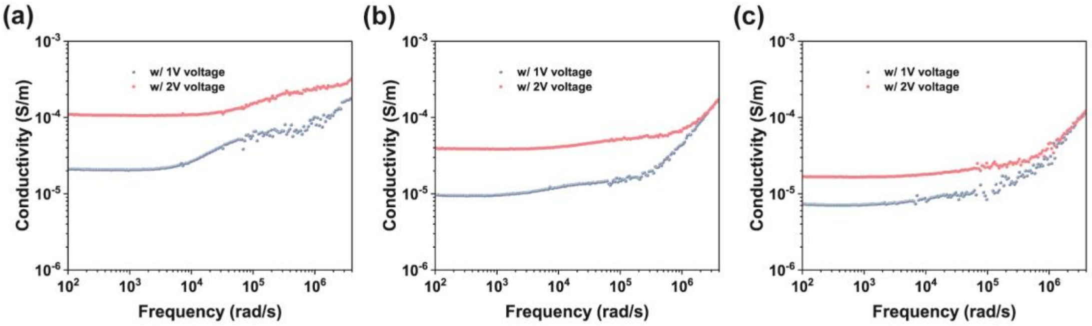

Supplementary Fig. 2. Conductivity–frequency plots of D18-based devices. (a) D18:BTP-eC9. (b) D18:IT-4F. (c) D18:L8-BO.

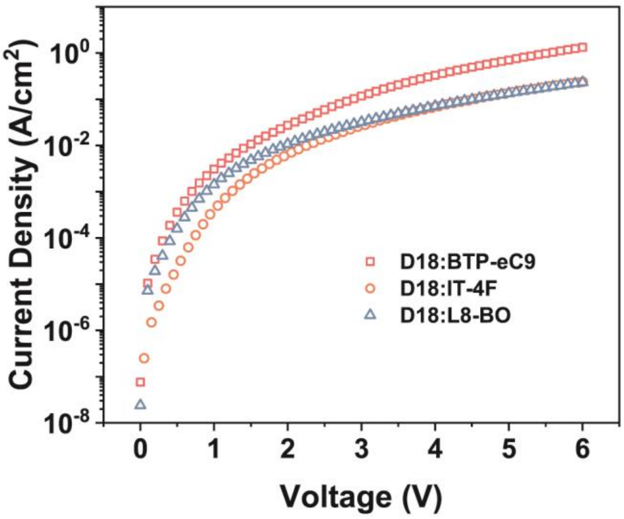

Supplementary Fig. 3. Current density–voltage (*J*–*V*) characteristics of D18-based devices.

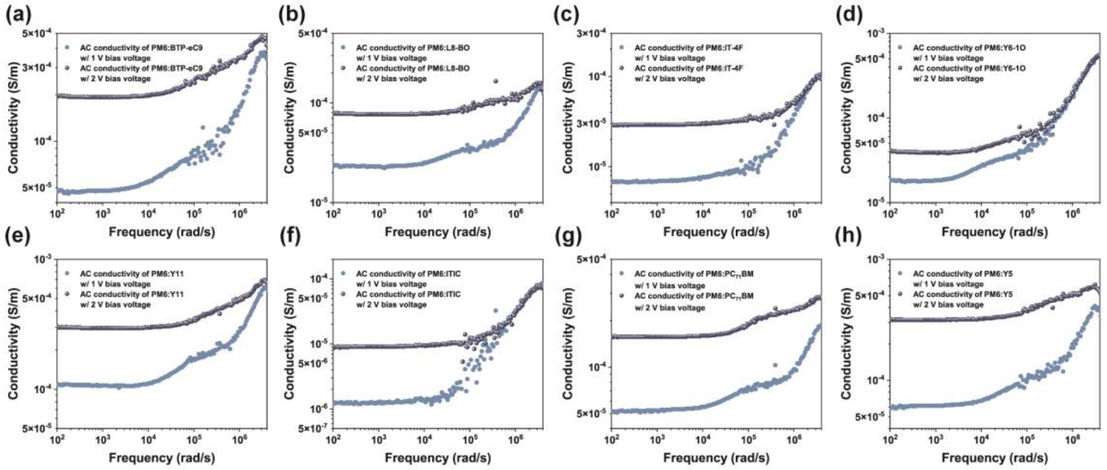

Supplementary Fig. 4. AC conductivity diagrams of different systems at 1 V and 2 V voltages. (a) PM6:BTP-eC9. (b) PM6:L8-BO. (c) PM6:IT-4F. (d) PM6:Y6-1O. (e) PM6:Y11. (f) PM6:ITIC. (g) PM6:$PC_{71}BM$. (h) PM6:Y5.

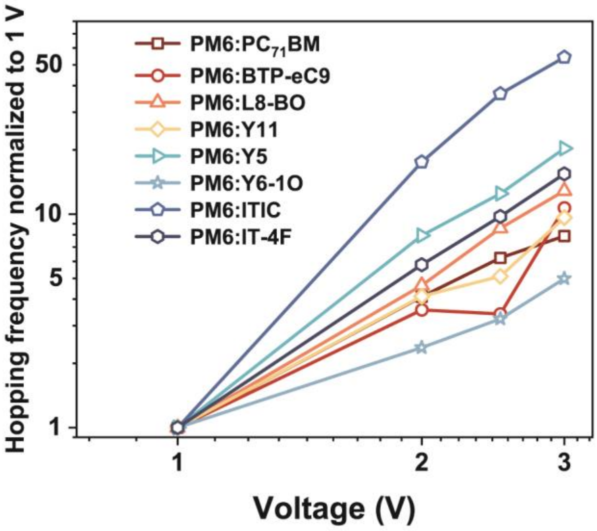

Supplementary Fig. 5. The voltage dependence of charge carrier hopping frequency, normalized to the hopping frequency at 1 V voltage.

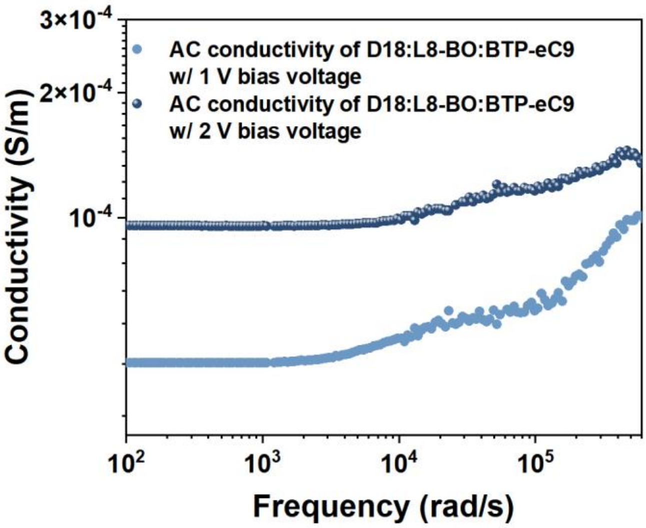

Supplementary Fig. 6. AC conductivity diagrams of D18:L8-BO:BTP-eC9 at 1 V and 2 V voltages.

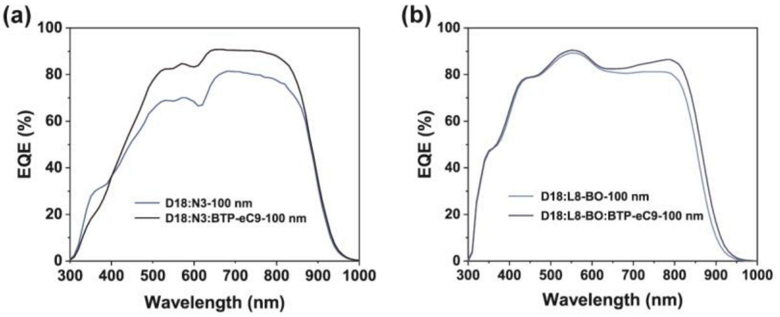

Supplementary Fig. 7. The external quantum efficiency (EQE) spectra. (a) EQE spectra of D18:N3 system. (b) EQE spectra of D18:L8-BO system.

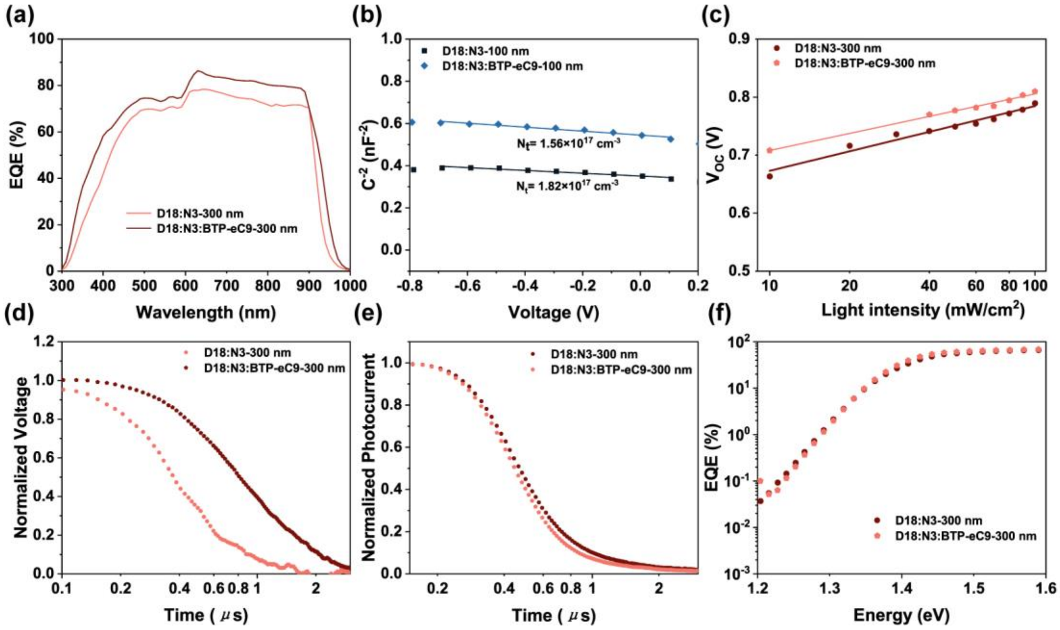

Supplementary Fig. 8. Charge extraction, recombination, and defect state analysis. (a) EQE spectra of devices based on D18:N3 system. (b) Capacitance (*C*) versus voltage (*V*) measurements and extracted C^{-2} versus *V* plots at 10 kHz. (c) The dependence of open-circuit voltage on light intensity. (d) Normalized transient photovoltaic (TPV) data for the devices based on D18:N3 system. (e) Normalized transient photocurrent (TPC) data for the devices based on D18:N3 system. (f) Fourier transform photocurrent spectroscopy (FTPS) data for the devices based on D18:N3 system.

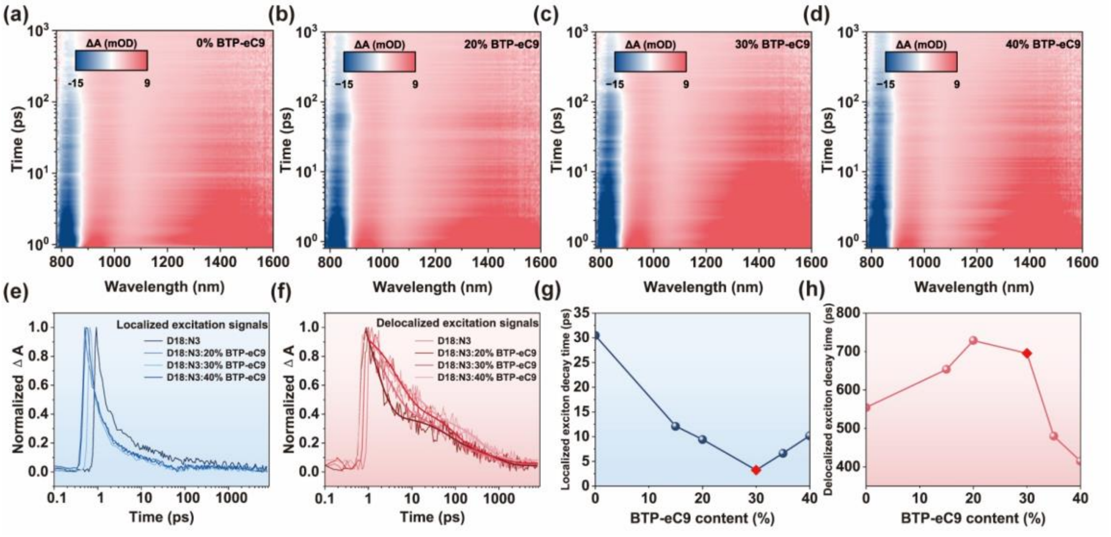

Supplementary Fig. 9. Femtosecond resolved transient absorption spectroscopy (TAS) results of D18:N3 system. (a) 2D TA data of D18:N3. (b) 2D TA data of D18:N3:20.0% BTP-eC9 content. (c) 2D TA data D18:N3:30.0% BTP-eC9. (d) 2D TA data D18:N3:40.0% BTP-eC9. (e) Decay dynamics probed at 912 nm from the active layers with different BTP-eC9 contents. (f) Decay dynamics probed at 1432 nm from the active layers with different BTP-eC9 contents. (g) Average decay time of localized excitons in the active layers with different BTP-eC9 contents. (h) Average decay time of delocalized excitons in the active layers with different BTP-eC9 contents.

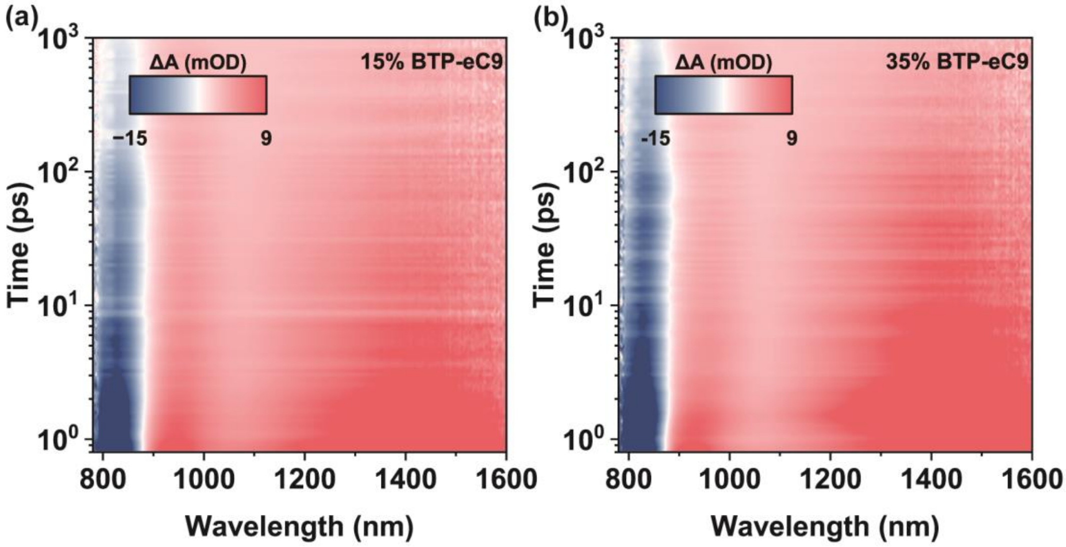

Supplementary Fig. 10. 2D TA spectra. (a) D18:N3:15.0% BTP-eC9. (b) D18:N3:35.0% BTP-eC9.

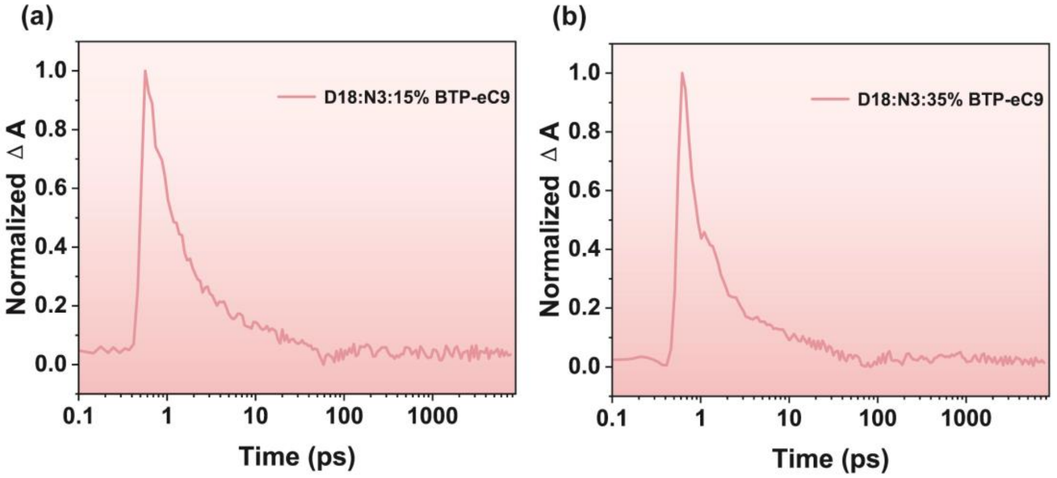

Supplementary Fig. 11. Localized exciton signals in D18:N3 systems with different proportions of BTP-eC9. (a) D18:N3:15.0% BTP-eC9. (b) D18:N3:35.0% BTP-eC9.

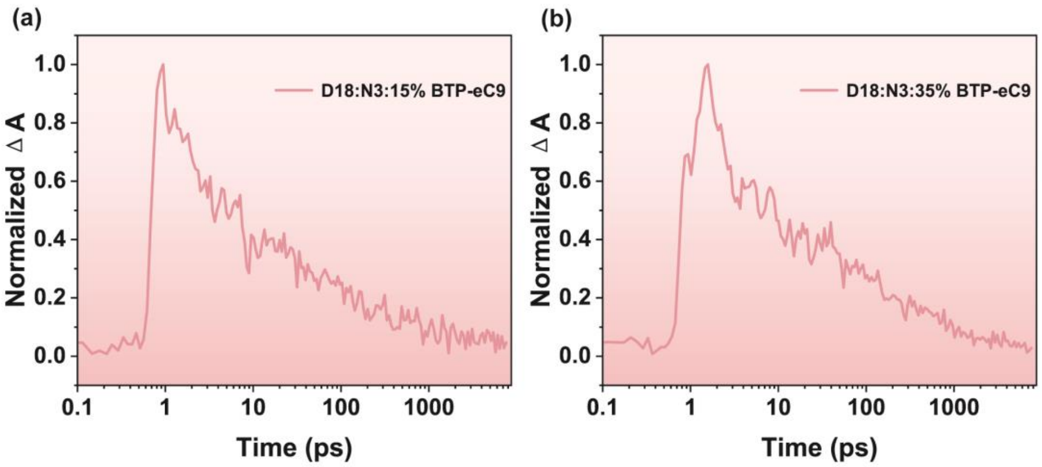

Supplementary Fig. 12. Delocalized exciton signals in D18:N3 systems with different proportions of BTP-eC9. (a) D18:N3:15.0% BTP-eC9. (b) D18:N3:35.0% BTP-eC9.

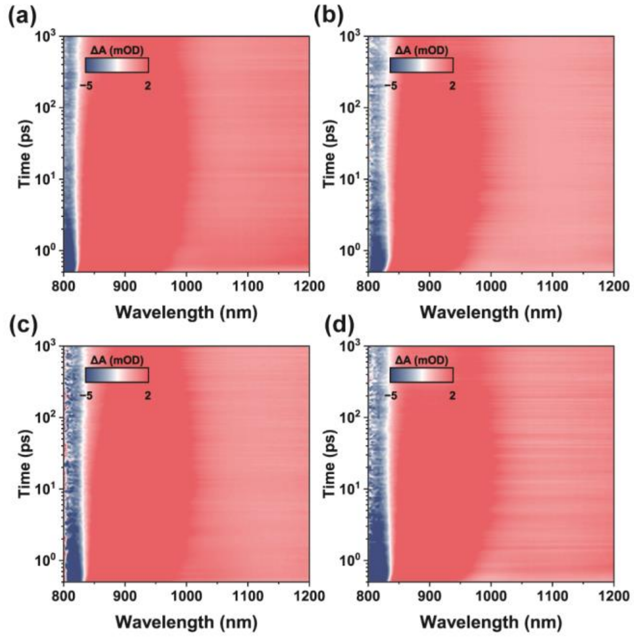

Supplementary Fig. 13. 2D TA spectra. (a) D18:L8-BO:33.3% BTP-eC9. (b) D18:L8-BO:50.0% BTP-eC9. (c) D18:L8-BO:66.7% BTP-eC9. (d) D18:L8-BO:83.3% BTP-eC9.

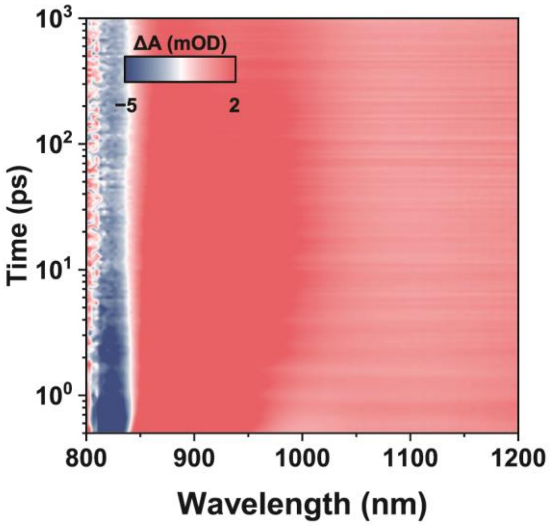

Supplementary Fig. 14. 2D TA spectra of D18:BTP-eC9.

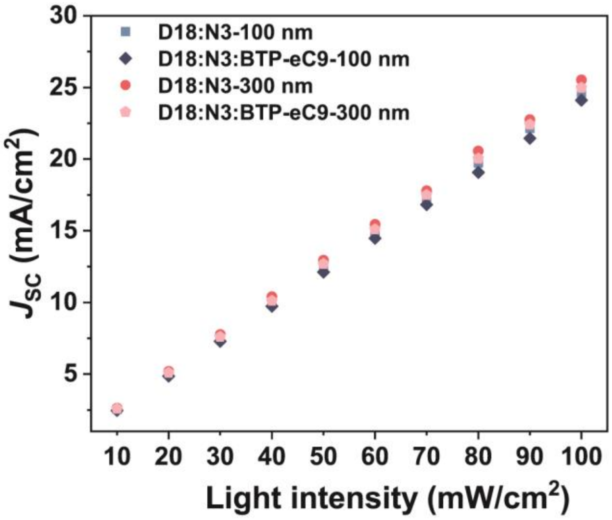

Supplementary Fig. 15. The dependence of short-circuit current density (J_{SC}) on light intensity.

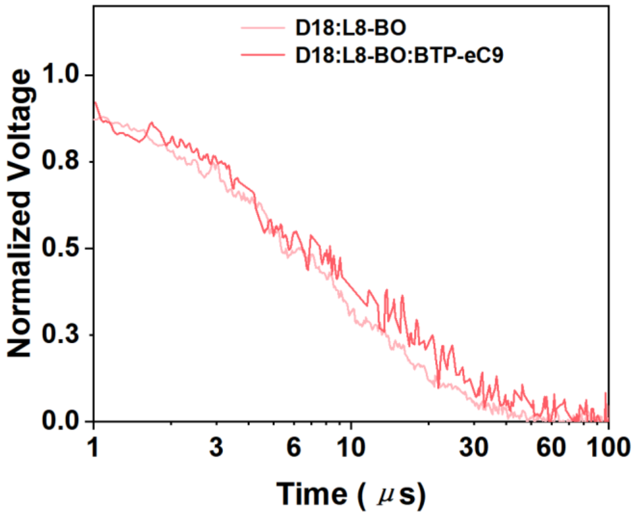

Supplementary Fig. 16. Normalized TPV data for the devices based on D18:L8-BO system.

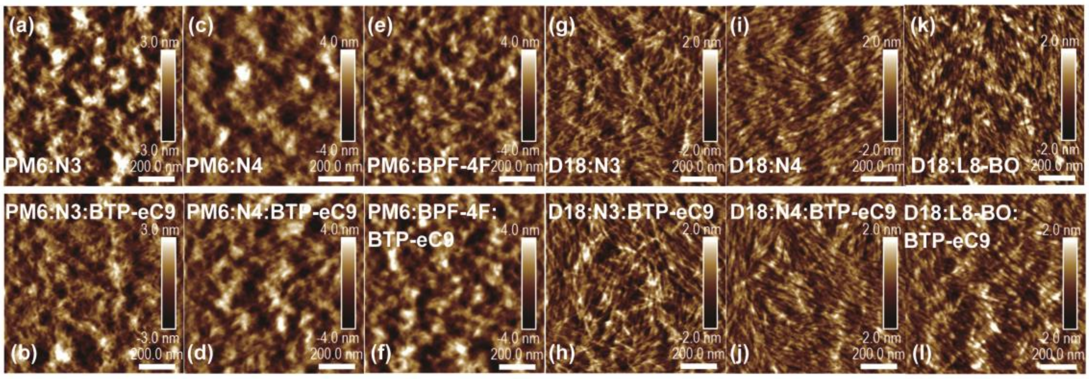

Supplementary Fig. 17. AFM image of active layer with a thickness of 100 nm. (a) PM6:N3. (b) PM6:N3:BTP-eC9. (c) PM6:N4. (d) PM6:N4:BTP-eC9. (e) PM6:BPF-4F. (f) PM6:BPF-4F:BTP-eC9. (g) D18:N3. (h) D18:N3:BTP-eC9. (i) D18:N4. (j) D18:N4:BTP-eC9. (k) D18:L8-BO. (l) D18:L8-BO:BTP-eC9.

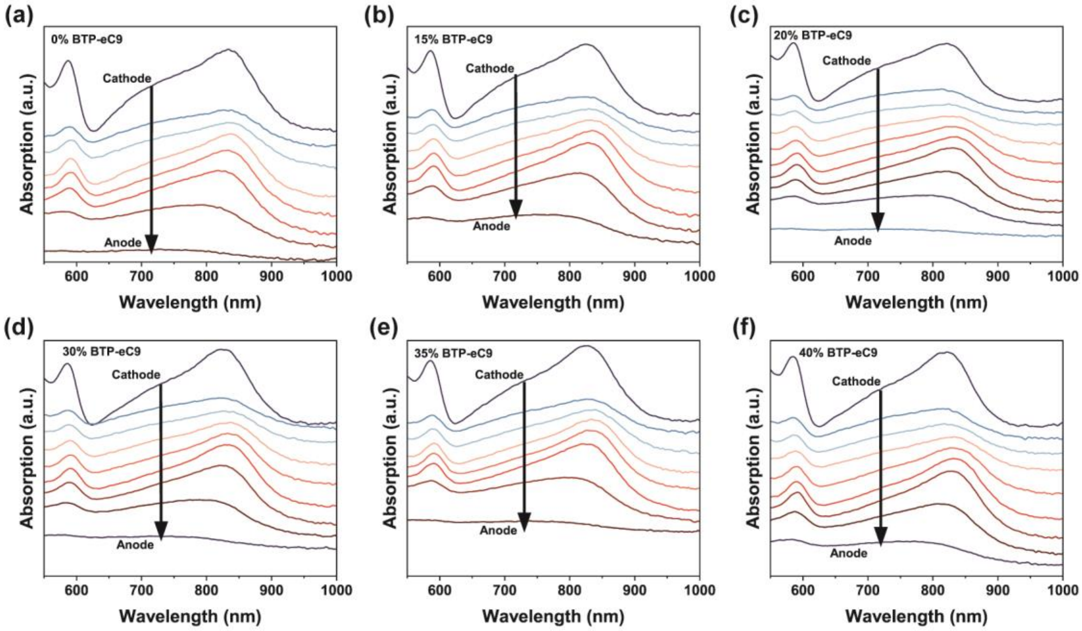

Supplementary Fig. 18. Diagrams of offset sublayers in the study of the film depth–dependent light absorption spectroscopy (FLAS). (a) D18:N3. (b) D18:N3:15.0% BTP-eC9. (c) D18:N3:20.0% BTP-eC9. (d) D18:N3:30.0% BTP-eC9. (e) D18:N3:35.0% BTP-eC9. (f) D18:N3:40.0% BTP-eC9.

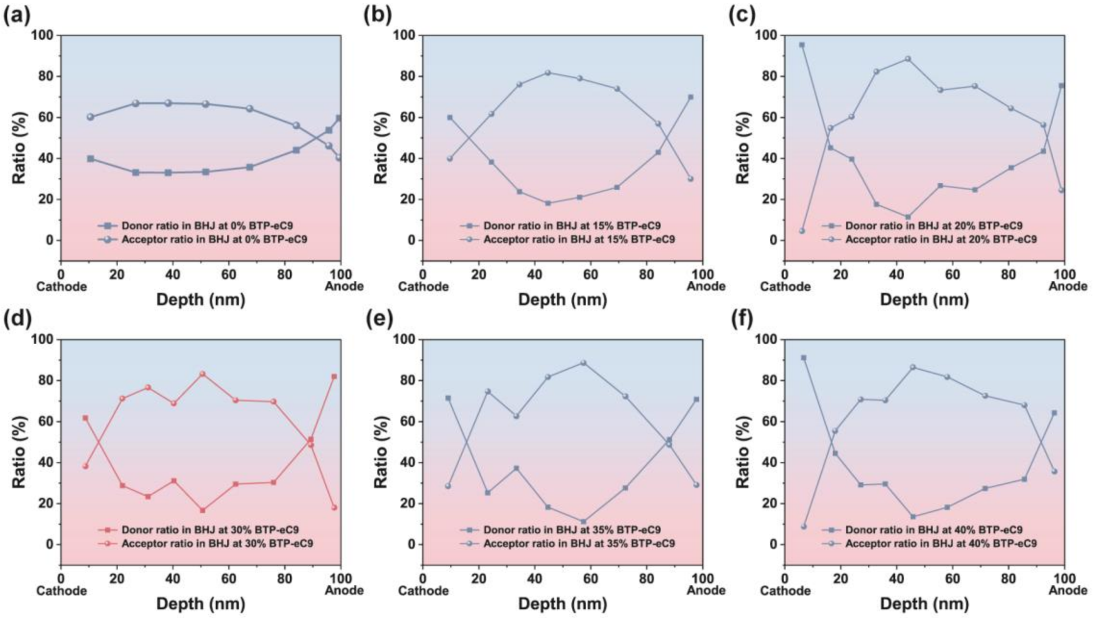

Supplementary Fig. 19. Distribution of donors and acceptors in the vertical direction in the D18:N3 system with different proportions of BTP-eC9. (a) D18:N3. (b) D18:N3:15.0% BTP-eC9. (c) D18:N3:20.0% BTP-eC9. (d) D18:N3:30.0% BTP-eC9. (e) D18:N3:35.0% BTP-eC9. (f) D18:N3:40.0% BTP-eC9.

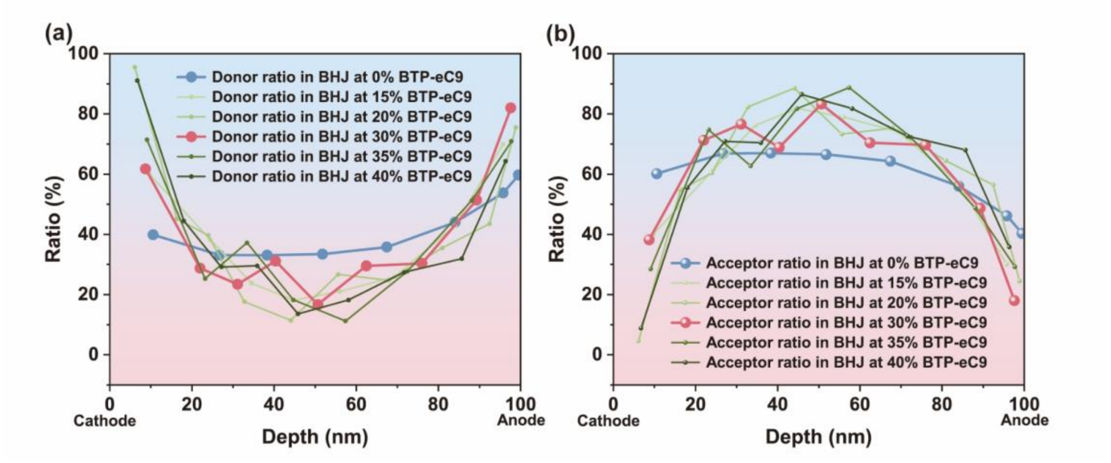

Supplementary Fig. 20. Comparison of the distribution of donors and acceptors in the vertical direction in the D18:N3 system with different proportions of BTP-eC9. (a) Distribution diagram of the donor in the vertical direction. (b) Distribution diagram of the acceptor in the vertical direction.

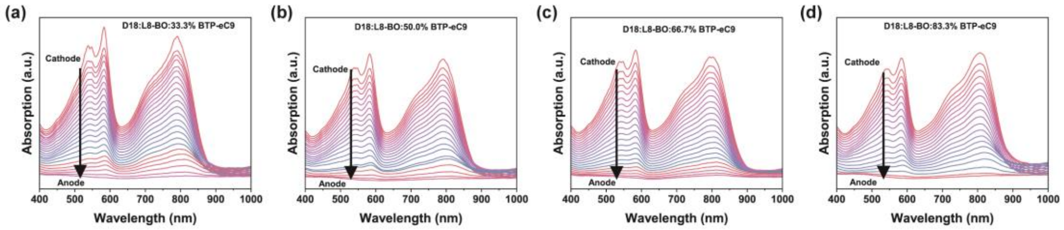

Supplementary Fig. 21. Film depth–dependent light absorption spectroscopy (FLAS) of the D18: L8-BO system with different proportions of BTP-eC9. (a) D18:L8-BO:33.3% BTP-eC9. (b) D18:L8-BO:50.0% BTP-eC9. (c) D18:L8-BO:66.7% BTP-eC9. (d) D18:L8-BO:83.3% BTP-eC9.

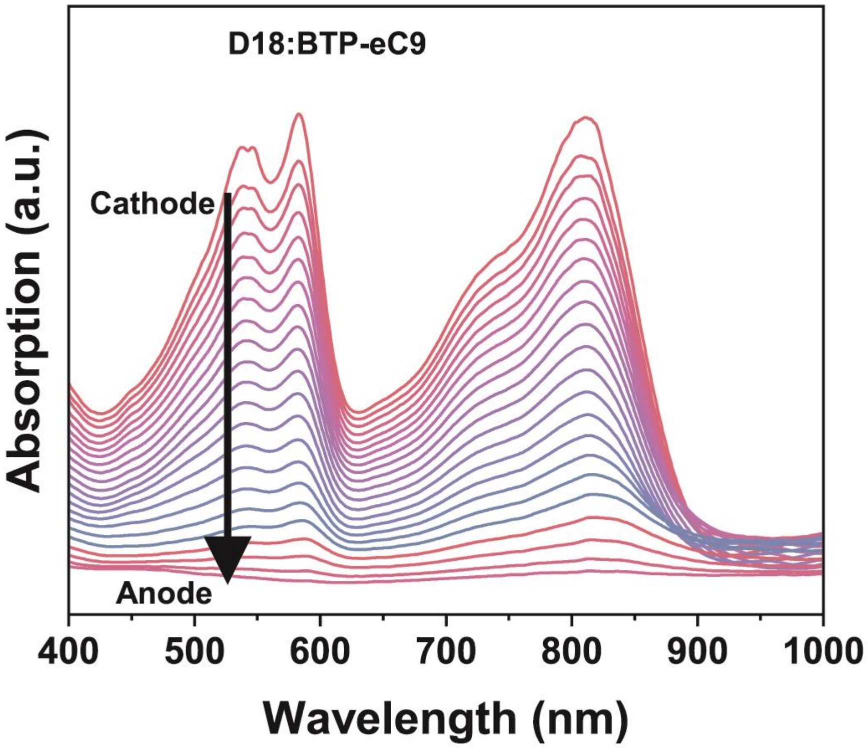

Supplementary Fig. 22. Film depth–dependent light absorption spectroscopy (FLAS) of the D18:BTP-eC9 system.

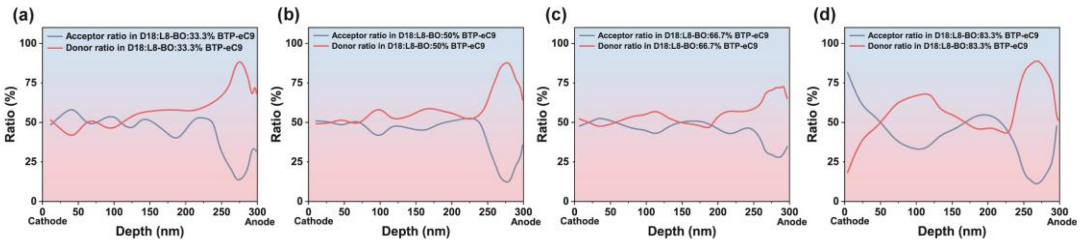

Supplementary Fig. 23. Distribution of donors and acceptors in the vertical direction in the D18:L8-BO system with different proportions of BTP-eC9. (a) D18:L8-BO:33.3% BTP-eC9. (b) D18:L8-BO:50.0% BTP-eC9. (c) D18:L8-BO:66.7% BTP-eC9. (d) D18:L8-BO:83.3% BTP-eC9.

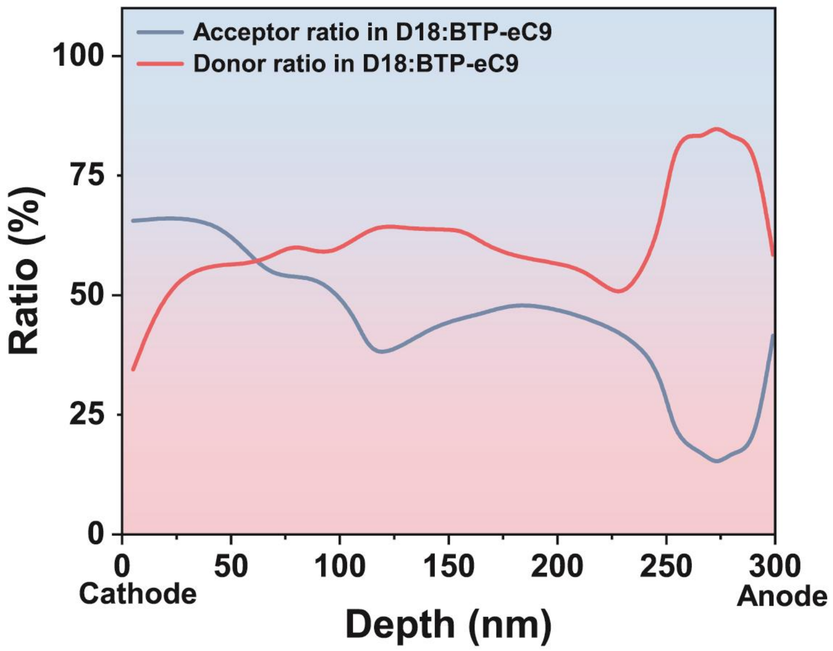

Supplementary Fig. 24. Distribution of donors and acceptors in the vertical direction in the D18:BTP-eC9 system.

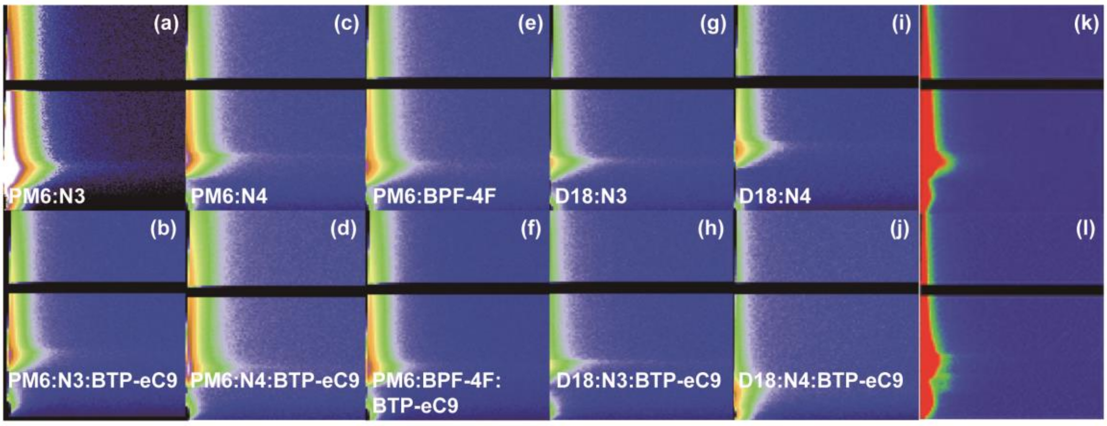

Supplementary Fig. 25. Grazing incidence small angle X-ray scattering (GISAXS) image of active layer with a thickness of 100 nm. (a) PM6:N3. (b) PM6:N3:BTP-eC9. (c) PM6:N4. (d) PM6:N4:BTP-eC9. (e) PM6:BPF-4F. (f) PM6:BPF-4F:BTP-eC9. (g) D18:N3. (h) D18:N3:BTP-eC9. (i) D18:N4. (j) D18:N4:BTP-eC9.

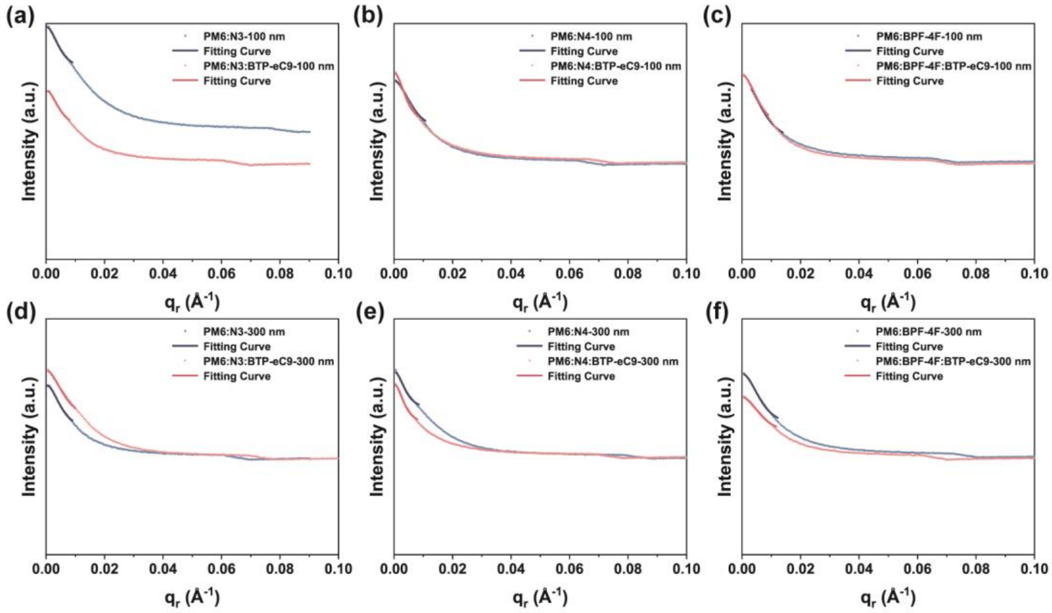

Supplementary Fig. 26. Debye-Anderson Brumberger (DAB) fitting of one-dimensional lines extracted from 2D GISAXS images. (a) PM6:N3 and PM6:N3:BTP-eC9 systems with an active layer thickness of 100 nm. (b) PM6:N4 and PM6:N4:BTP-eC9 systems with an active layer thickness of 100 nm. (c) PM6:BPF-4F and PM6:BPF-4F:BTP-eC9 systems with an active layer thickness of 100 nm. (d) PM6:N3 and PM6:N3:BTP-eC9 systems with an active layer thickness of 300 nm. (e) PM6:N4 and PM6:N4:BTP-eC9 systems with an active layer thickness of 300 nm. (f) PM6:BPF-4F and PM6:BPF-4F:BTP-eC9 systems with an active layer thickness of 300 nm.

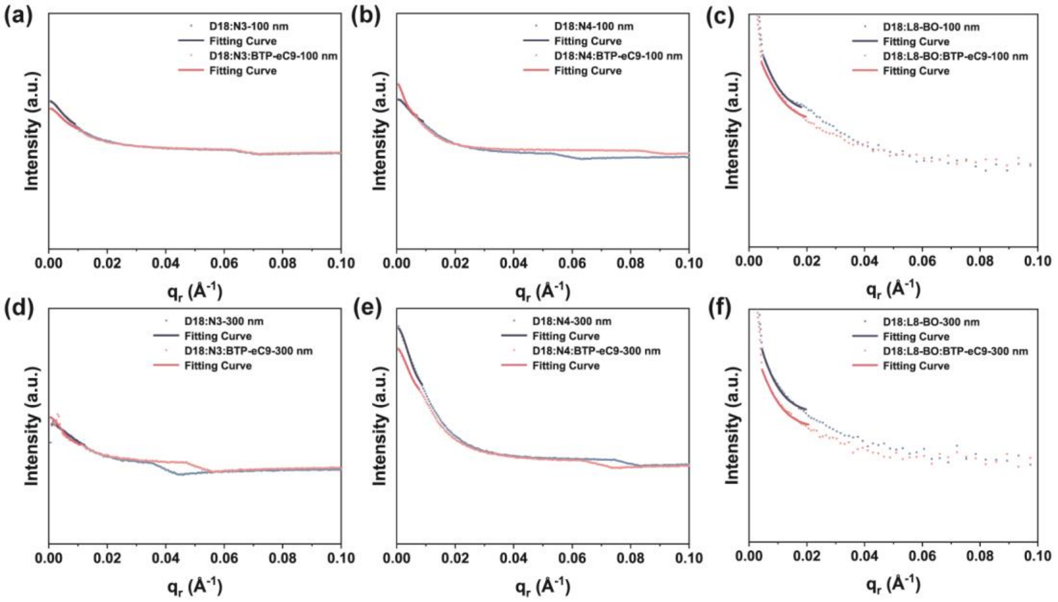

Supplementary Fig. 27. DAB fitting of one-dimensional lines extracted from 2D GISAXS images. (a) D18:N3 and D18:N3:BTP-eC9 systems with an active layer thickness of 100 nm. (b) D18:N4 and D18:N4:BTP-eC9 systems with an active layer thickness of 100 nm. (c) D18:L8-BO and D18:L8-BO:BTP-eC9 systems with an active layer thickness of 100 nm. (d) D18: N3 and D18:N3:BTP-eC9 systems with an active layer thickness of 300 nm. (e) D18:N4 and D18:N4:BTP-eC9 systems with an active layer thickness of 300 nm. (f) D18:L8-BO and D18:L8-BO:BTP-eC9 systems with an active layer thickness of 300 nm.

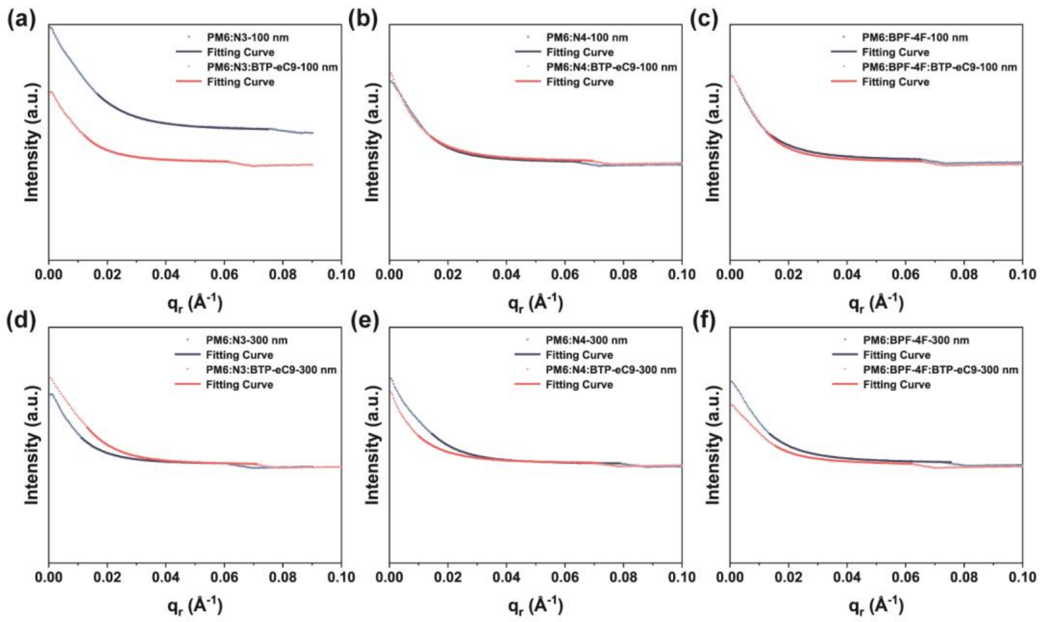

Supplementary Fig. 28. Fractal fitting of one-dimensional lines extracted from 2D GISAXS images. (a) PM6:N3 and PM6:N3:BTP-eC9 systems with an active layer thickness of 100 nm. (b) PM6:N4 and PM6:N4:BTP-eC9 systems with an active layer thickness of 100 nm. (c) PM6:BPF-4F and PM6:BPF-4F:BTP-eC9 systems with an active layer thickness of 100 nm. (d) PM6:N3 and PM6:N3:BTP-eC9 systems with an active layer thickness of 300 nm. (e) PM6:N4 and PM6:N4:BTP-eC9 systems with an active layer thickness of 300 nm. (f) PM6:BPF-4F and PM6:BPF-4F:BTP-eC9 systems with an active layer thickness of 300 nm.

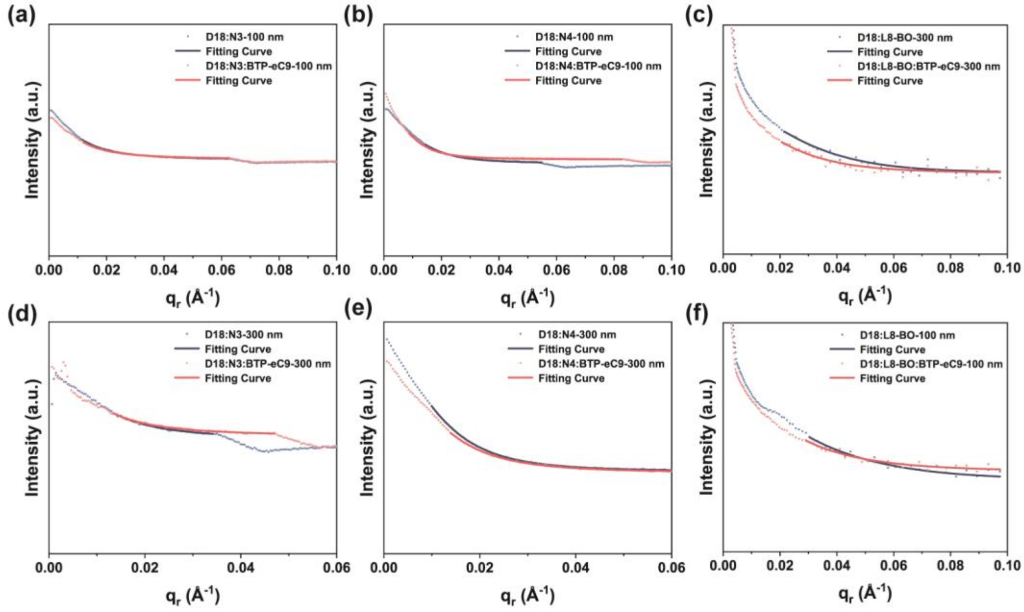

Supplementary Fig. 29. Fractal fitting of one-dimensional lines extracted from 2D GISAXS images. (a) D18:N3 and D18:N3:BTP-eC9 systems with an active layer thickness of 100 nm. (b) D18:N4 and D18:N4:BTP-eC9 systems with an active layer thickness of 100 nm. (c) D18:L8-BO and D18:L8-BO:BTP-eC9 systems with an active layer thickness of 100 nm. (d) D18: N3 and D18:N3:BTP-eC9 systems with an active layer thickness of 300 nm. (e) D18:N4 and D18:N4:BTP-eC9 systems with an active layer thickness of 300 nm. (f) D18:L8-BO and D18:L8-BO:BTP-eC9 systems with an active layer thickness of 300 nm.

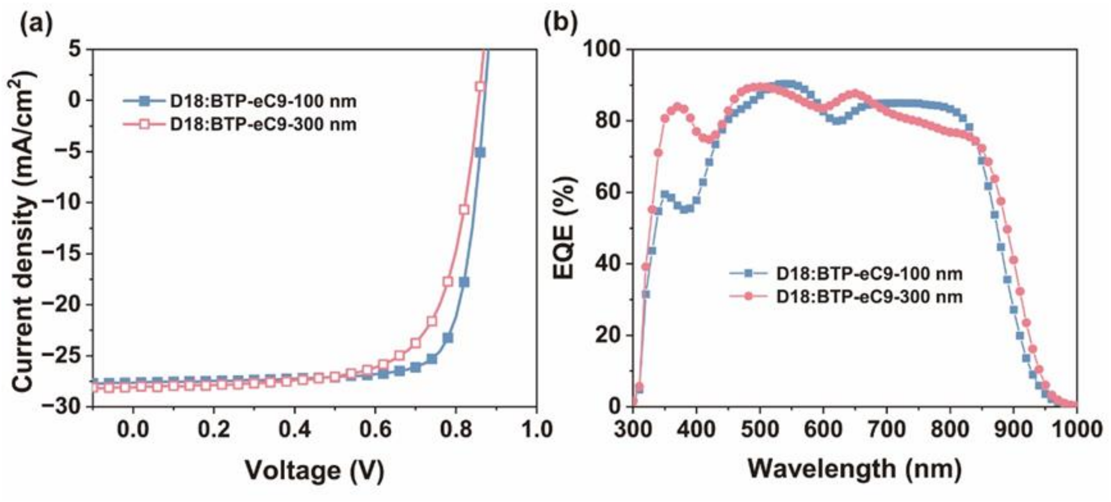

Supplementary Fig. 30. Current density–voltage (*J*–*V*) characteristics and external quantum efficiency (EQE) spectra of D18:BTP-eC9 devices. (a) *J*–*V* curve. (b) EQE spectrum.

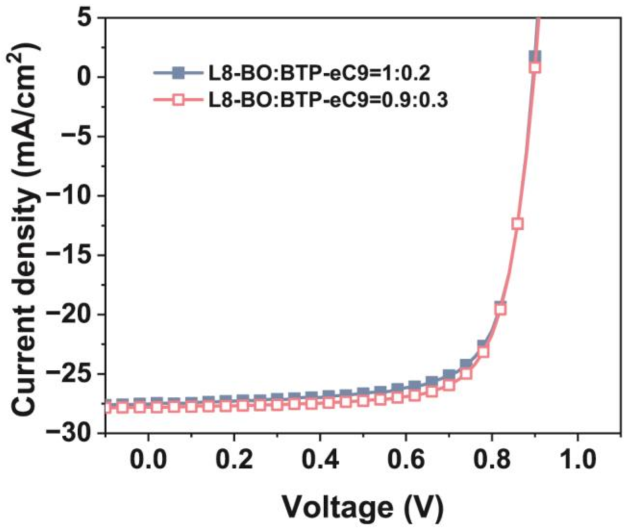

Supplementary Fig. 31. Current density–voltage (*J*–*V*) curves of devices with varying BTP-eC9 ratios.

Supplementary Tables

Supplementary Table 1. Zero-field mobility (μ_0), field-dependent of mobility (β), hopping frequency (ω_H) and critical length (L_C) of D18-based systems.

	μ_0 ($cm^2V^{-1}s^{-1}$)	β ($cm^{1/2}V^{-1/2}$)	ω_H with 1 V voltage (rad/s)	ω_H with 2 V voltage (rad/s)	L_C (nm)
**D18:BTP-eC9**	1.50×10^{-4}	7.79×10^{-3}	3.07×10^{5}	3.34×10^{5}	40.19
**D18:IT-4F**	5.99×10^{-5}	5.79×10^{-3}	2.83×10^{5}	1.55×10^{6}	31.63
**D18:L8-BO**	7.50×10^{-5}	5.13×10^{-3}	3.67×10^{5}	8.44×10^{5}	38.97

Supplementary Table 2. Hopping frequency under different voltages.

	At 1 V (rad/s)	At 2 V (rad/s)	At 2.5 V (rad/s)	At 3 V (rad/s)
PM6:$PC_{71}BM$	1.09×10^6	4.47×10^6	6.79×10^6	8.60×10^6
PM6:BTP-eC9	3.52×10^5	1.25×10^6	1.20×10^6	3.76×10^6
PM6:L8-BO	6.41×10^5	2.97×10^6	5.50×10^6	8.24×10^6
PM6:Y11	4.69×10^5	1.93×10^6	2.40×10^6	4.51×10^6
PM6:Y5	3.16×10^5	2.51×10^6	3.94×10^6	6.41×10^6
PM6:Y6-1O	1.04×10^5	2.46×10^5	3.36×10^5	5.19×10^5
PM6:ITIC	2.56×10^4	4.48×10^5	9.37×10^5	1.39×10^6
PM6:IT-4F	2.26×10^5	1.31×10^6	2.20×10^6	3.49×10^6
D18:L8-BO:BTP-eC9	3.97×10^5	2.30×10^6	2.94×10^6	4.39×10^6

Supplementary Table 3. Zero-field mobility (μ_0), field-dependent of mobility (β), and critical length (L_C) of different systems.

	μ_0 ($\times 10^{-4}$ cm^{2}V^{-1}s^{-1})	β ($\times 10^{-2}$ cm$^{1/2}$V$^{-1/2}$)	L_C (nm)
PM6:BTP-eC9	0.80	0.55	37.60
PM6:Y11	0.47	0.49	20.85
PM6:Y6-1O	0.16	0.50	30.71
PM6:$PC_{71}BM$	1.36	0.84	8.93
PM6:ITIC	0.05	0.81	15.09
PM6:IT-4F	0.45	0.55	32.31
PM6:Y5	0.59	1.07	8.15
PM6:L8-BO	1.41	0.54	37.21
D18:L8-BO:BTP-eC9	1.92	0.62	62.82

Supplementary Table 4. Average decay time of localized excitation signal of D18:L8-BO system.

	A_1 (%)	t_1	A_2 (%)	t_2	Average time (ps)
D18:L8-BO	1.90	0.90	0.33	43.47	38.89
D18: L8-BO:8.3% BTP-eC9	1.51	1.34	0.63	24.90	22.23
D18: L8-BO:33.3% BTP-eC9	2.62	0.73	0.40	44.78	40.46
D18: L8-BO:50.0% BTP-eC9	3.90	0.49	0.39	44.49	40.21
D18: L8-BO:66.7% BTP-eC9	3.33	0.65	0.53	49.43	42.71
D18: L8-BO:83.3% BTP-eC9	2.78	0.51	0.32	41.84	37.85
D18:BTP-eC9	6.01	0.33	0.41	32.00	27.81

Supplementary Table 5. Average decay time of localized excitation signal of D18:N3 system.

	A_1 (%)	t_1	A_2 (%)	t_2	A_3 (%)	t_3	Average time (ps)
D18:N3	0.52	3.01	10.85	0.35	0.18	48.79	30.46
D18:N3:15% BTP-eC9	0.69	1.41	2.9	0.36	0.22	17.92	12.13
D18:N3:20% BTP-eC9	1.82	0.82	19.74	0.14	0.27	17.68	9.43
D18:N3:30% BTP-eC9	2.01	0.92	1.61	0.14	0.28	18.17	3.37
D18:N3:35% BTP-eC9	1.39	1.07	48.61	0.15	0.29	17.55	6.63
D18:N3:40% BTP-eC9	0.87	1.29	8.53	0.26	0.26	17.29	10.19

Supplementary Table 6. Average decay time of delocalized excitation signal of D18:N3 system.

	A_1 (%)	t_1	A_2 (%)	t_2	A_3 (%)	t_3	Average time (ps)
D18:N3	1.09	2.93	0.43	62.05	0.25	646.76	554.56
D18:N3:15% BTP-eC9	0.96	3.13	0.55	75.41	0.27	1373.88	653.31
D18:N3:20% BTP-eC9	2.33	1.24	0.37	65.59	0.30	802.47	728.36
D18:N3:30% BTP-eC9	1.27	4.66	0.67	97.60	0.25	890.49	695.32
D18:N3:35% BTP-eC9	6.05	0.85	0.61	25.77	0.59	511.46	479.98
D18:N3:40% BTP-eC9	0.96	1.62	0.56	17.99	0.58	433.41	414.74

Supplementary Table 7. Hole transfer rate of D18:L8-BO system.

	A_1 (%)	t_1	A_2 (%)	t_2	Hole transfer rate (ps^{-1})
D18:L8-BO	3.21	0.27	0.33	2.35	0.80
D18: L8-BO:8.3% BTP-eC9	7.04	0.30	0.38	2.09	1.27

Supplementary Table 8. The relevant numerical values used for calculating defect states.

	Slope	Relative permittivity	Trap density (cm^{-3})
D18:N3	-0.066	2.94	1.82×10^{17}
D18:N3:BTP-eC9	-0.094	2.42	1.56×10^{17}
D18:L8-BO	-0.20	2.96	6.17×10^{16}
D18:L8-BO: BTP-eC9	-0.26	2.80	4.94×10^{16}

Supplementary Table 9. Light intensity dependence of short-circuit current density (α).

	α
D18:N3-100 nm	0.99
D18:N3:BTP-eC9-100 nm	0.99
D18:N3-300 nm	0.99
D18:N3:BTP-eC9-300 nm	0.99
D18:L8-BO-300 nm	0.97
D18:L8-BO:BTP-eC9-300 nm	0.99

Supplementary Table 10. Light intensity dependence of open-circuit voltage (n).

	n
D18:N3-300 nm	1.87
D18:N3:BTP-eC9-300 nm	1.64
D18:L8-BO-300 nm	1.89
D18:L8-BO:BTP-eC9-300 nm	1.70

Supplementary Table 11. Charge carrier extraction time from transient photocurrent (TPC) measurements.

	Charge carrier extraction time (μs)
D18:N3-300 nm	0.28
D18:N3:BTP-eC9-300 nm	0.24
D18:L8-BO-300 nm	0.93
D18:L8-BO:BTP-eC9-300 nm	0.43

Supplementary Table 12. Carrier lifetime in D18:L8-BO system.

	Carrier lifetime (μs)
D18:L8-BO-300 nm	10.45
D18:L8-BO:BTP-eC9-300 nm	15.29

Supplementary Table 13. Urbach energy in devices.

	Urbach energy (meV)
D18:N3-300 nm	26.5
D18:N3:BTP-eC9-300 nm	24.8
D18:L8-BO-300 nm	23.0
D18:L8-BO:BTP-eC9-300 nm	20.8

Supplementary Table 14. The intermixing domain size (ξ) in the system with PM6 as the donor.

	ξ (nm)
PM6:N3-100 nm	20.98
PM6:N3:BTP-eC9-100 nm	20.27
PM6:N3-300 nm	19.27
PM6:N3:BTP-eC9-300 nm	16.96
PM6:N4-100 nm	17.22
PM6:N4:BTP-eC9-100 nm	25.69
PM6:N4-300 nm	23.71
PM6:N4:BTP-eC9-300 nm	26.04
PM6:BPF-4F-100 nm	15.93
PM6:BPF-4F:BTP-eC9-100 nm	18.72
PM6:BPF-4F-300 nm	16.87
PM6:BPF-4F:BTP-eC9-300 nm	1.43

Supplementary Table 15. The intermixing domain size (ξ) in the system with D18 as the donor.

	ξ (nm)
D18:N3-100 nm	16.01
D18:N3:BTP-eC9-100 nm	15.01
D18:N3-300 nm	9.42
D18:N3:BTP-eC9-300 nm	14.95
D18:N4-100 nm	16.36
D18:N4:BTP-eC9-100 nm	31.23
D18:N4-300 nm	19.55
D18:N4:BTP-eC9-300 nm	18.70
D18:L8-BO-100 nm	30.75
D18:L8-BO:BTP- eC9-100 nm	23.37
D18:L8-BO-300 nm	39.61
D18:L8-BO:BTP- eC9-300 nm	27.07

Supplementary Table 16. The acceptor domain size in the system with PM6 as the donor. The correlation length and fractal dimension of the fractal-like acceptor aggregates are denoted by η and D, respectively. The Guinier radius (R_g) is used to characterize the average domain size of the acceptor phase.

	D	η (nm)	R_g (nm)
PM6:N3-100 nm	2.61	19.14	41.54
PM6:N3:BTP-eC9-100 nm	2.44	20.60	42.24
PM6:N3-300 nm	2.42	14.56	29.62
PM6:N3:BTP-eC9-300 nm	2.70	14.58	32.54
PM6:N4-100 nm	2.50	17.88	37.34
PM6:N4:BTP-eC9-100 nm	2.31	19.76	38.60
PM6:N4-300 nm	2.60	14.19	30.72
PM6:N4:BTP-eC9-300 nm	2.13	17.68	32.29
PM6:BPF-4F-100 nm	2.30	18.21	35.41
PM6:BPF-4F:BTP-eC9-100 nm	2.49	23.30	48.71
PM6:BPF-4F-300 nm	2.31	20.14	39.31
PM6:BPF-4F:BTP-eC9-300 nm	2.32	20.78	40.77

Supplementary Table 17. The acceptor domain size in the system with D18 as the donor. The correlation length and fractal dimension of the fractal-like acceptor aggregates are denoted by η and D, respectively. The Guinier radius (R_g) is used to characterize the average domain size of the acceptor phase.

	D	η (nm)	R_g (nm)
D18:N3-100 nm	2.12	13.69	24.87
D18:N3:BTP-eC9-100 nm	2.04	15.67	27.54
D18:N3-300 nm	2.10	12.49	22.49
D18:N3:BTP-eC9-300 nm	2.00	15.94	27.60
D18:N4-100 nm	2.62	9.38	20.44
D18:N4:BTP-eC9-100 nm	2.62	13.50	29.40
D18:N4-300 nm	2.67	14.31	31.67
D18:N4:BTP-eC9-300 nm	2.60	12.65	27.37
D18:L8-BO-100 nm	2.92	17.87	42.73
D18:L8-BO:BTP-eC9-100 nm	2.80	21.57	49.79
D18:L8-BO-300 nm	2.96	6.87	16.62
D18:L8-BO:BTP-eC9-300 nm	2.78	8.76	20.10

Supplementary Table 18. Device performance of D18:BTP-eC9.

	Thickness (nm)	V_{OC} (V)	J_{SC}/J_{EQE} (mA/cm^2)	FF (%)	PCE (%)
**D18:BTP-eC9**	100	0.87	27.63/26.92	77.9	18.7
	300	0.86	28.10/27.67	69.3	16.7

Supplementary Table 19. Device performance of 300 nm-thick D18:L8-BO:BTP-eC9 devices.

	Ratio	V_{OC} (V)	J_{SC}	FF (%)	PCE (%)
**D18:L8-BO:BTP-eC9**	1:1.1:0.1	0.90	27.88	75.4	19.0
	1:1:0.2	0.90	27.79	74.0	18.5
	1:0.9:0.3	0.90	27.55	72.8	18.0

Supplementary References

1. Proctor, C. M., Kim, C., Neher, D. & Nguyen, T.-Q. Nongeminate Recombination and Charge Transport Limitations in Diketopyrrolopyrrole-Based Solution-Processed Small Molecule Solar Cells. *Adv. Funct. Mater.* **23**, 3584–3594 (2013).
2. Frenkel, J. On Pre-Breakdown Phenomena in Insulators and Electronic Semi-Conductors. *Phys. Rev.* **54**, 647–648 (1938).
3. Novikov, S. V. Charge Carrier Diffusion in Energy Landscape Created by Static Charges: Poole–Frenkel Model Revised. *Phys. Status Solidi B* **236**, 119–128 (2003).
4. Sun, F. *et al.* 1,5-Diiodocycloctane: A Cyclane Solvent Additive That Can Extend the Exciton Diffusion Length in Thick Film Organic Solar Cells. *Energy Environ. Sci.* **17**, 1916–1930 (2024).
5. Xu, T. *et al.* 15.8% Efficiency Binary All-Small-Molecule Organic Solar Cells Enabled by a Selenophene Substituted Sematic Liquid Crystalline Donor. *Energy Environ. Sci.* **14**, 5366–5376 (2021).
6. Zou, W. *et al.* A Bithiazole-Substituted Donor for High-Efficiency Thick Ternary Organic Solar Cells via Regulation of Crystallinity and Miscibility. *Adv. Energy Mater.* **13**, 2300784 (2023).
7. Guo, C. *et al.* A Polycrystalline Polymer Donor as Pre-Aggregate toward Ordered Molecular Aggregation for 19.3% Efficiency Binary Organic Solar Cells. *Adv. Mater.* **35**, 2304921 (2023).

8. Yi, X. *et al.* Additive-Assisted Molecular Aggregation Manipulation Towards Efficient Thick Organic Solar Cells. *J. Mater. Chem. C* **12**, 17078–17088 (2024).
9. Gao, S. *et al.* Ameliorated Trap Density and Energetic Disorder Via a Strengthened Intermolecular Interaction Strategy to Construct Efficient Non-Halogenated Organic Solar Cells. *Energy Environ. Sci.* **17**, 5542–5551 (2024).
10. Lan, A. *et al.* Asymmetric Non-Fullerene Acceptor Derivatives Incorporated Ternary Organic Solar Cells. *ACS Appl. Mater. Interfaces* **15**, 39657–39668 (2023).
11. Desta Fenta, A., Lin, C.-W., Li, S.-W., Chen, C.-T. & Chen, C.-T. Difluoro and Dicyano Substituted Sexithiophene − Isoindigo Terpolymers Enabling Ternary Polymer Solar Cells with a Bulk Heterojunction Thickness 100 Nm → 300 Nm and a Minor Decline of Efficiency 14.5 % → 14.0 %. *Chem. Eng. J.* **468**, 143796 (2023).
12. Wang, X. *et al.* Dithienoquinoxaline-Quaterthiophene Wide Bandgap Donor Polymers with Strong Interchain Aggregation for Efficient Organic Solar Cells Processed with a Non-Halogenated Solvent. *J. Mater. Chem. A* **12**, 5731–5739 (2024).
13. Zhang, S. *et al.* Efficient Organic Solar Cells Enabled by Sustainable and Synergetic Device Engineering. *Chem. Eng. J.* **481**, 148728 (2024).
14. Fu, J. *et al.* Eutectic Phase Behavior Induced by a Simple Additive Contributes to Efficient Organic Solar Cells. *Nano Energy* **84**, 105862 (2021).
15. Jiang, D. *et al.* Extracting Charge Carrier Mobility in Organic Solar Cells Through

Space-Charge-Limited Current Measurements. *Mater. Sci. Eng. R Rep.* **157**, 100772 (2024).

16. Song, X. *et al.* Film-Formation Dynamics Coordinated by Intermediate State Engineering Enables Efficient Thickness-Insensitive Organic Solar Cells. *Energy Environ. Sci.* **16**, 3441–3452 (2023).
17. Zhang, Y. *et al.* Graded Bulk-Heterojunction Enables 17% Binary Organic Solar Cells Via Nonhalogenated Open Air Coating. *Nat. Commun.* **12**, 4815 (2021).
18. Fenta, A. D., Lu, C.-F., Gidey, A. T. & Chen, C.-T. High Efficiency Organic Photovoltaics with a Thick (300 nm) Bulk Heterojunction Comprising a Ternary Composition of a PFT Polymer–PC_{71} BM Fullerene–IT4F Nonfullerene Acceptor. *ACS Appl. Energy Mater.* **4**, 5274–5285 (2021).
19. Wei, Y. *et al.* High Performance As-Cast Organic Solar Cells Enabled by a Refined Double-Fibril Network Morphology and Improved Dielectric Constant of Active Layer. *Adv. Mater.* **36**, 2403294 (2024).
20. Yeop, J. *et al.* High-Crystalline Regioregular Polymer Semiconductor by Thermal Treatment for Thickness Tolerance Organic Photovoltaics. *Sol. RRL* **6**, 2200445 (2022).
21. Zhu, S. *et al.* High-Efficiency Thick Film Binary Organic Photovoltaics via Asymmetric Alkyl Chain Engineering. *Adv. Funct. Mater.* **34**, 2410786 (2024).
22. Zhao, H. *et al.* High-Performance Green Thick-Film Ternary Organic Solar Cells

Enabled by Crystallinity Regulation. *Adv. Funct. Mater.* **33**, 2210534 (2023).

23. Yoon, S. *et al.* High-Performance Scalable Organic Photovoltaics with High Thickness Tolerance from 1 cm^2 to Above 50 cm^2. *Joule* **6**, 2406–2422 (2022).
24. Liu, Z. & Wang, H.-E. High-Performance Ternary Organic Photovoltaics with $NC_{70}BA$ as the Third Component Material Enabling Thickness-Insensitive Photoactive Performance. *Nanotechnology* **33**, 065206 (2021).
25. Ho, C. H. Y. *et al.* Importance of Electric-Field-Independent Mobilities in Thick-Film Organic Solar Cells. *ACS Appl. Mater. Interfaces* **14**, 47961–47970 (2022).
26. Cai, Y. *et al.* Improved Molecular Ordering in a Ternary Blend Enables All-Polymer Solar Cells over 18% Efficiency. *Adv. Mater.* **35**, 2208165 (2023).
27. Zhao, H. *et al.* Kinetics Manipulation Enables High-Performance Thick Ternary Organic Solar Cells via R2R-Compatible Slot-Die Coating. *Adv. Mater.* **34**, 2105114 (2022).
28. Xu, J. *et al.* Modulating Aggregation Behavior by Ternary Strategies for Efficient and Stable Thick-Film Organic Solar Cells. *Small* **20**, 2406691 (2024).
29. Gao, J. *et al.* Over 16% Efficiency of Thick-Film Organic Photovoltaics with Symmetric and Asymmetric Non-Fullerene Materials as Alloyed Acceptor. *Sol. RRL* **5**, 2100365 (2021).
30. Wei, Y. *et al.* Over 18% Efficiency Ternary Organic Solar Cells with 300 nm Thick Active Layer Enabled by an Oligomeric Acceptor. *Adv. Mater.* **36**, 2304225 (2024).

31. Song, X. *et al.* Process-Aid Solid Engineering Triggers Delicately Modulation of Y-Series Non-Fullerene Acceptor for Efficient Organic Solar Cells. *Adv. Mater.* **34**, 2200907 (2022).

32. Dai, T. *et al.* Reduced Exciton Binding Energy and Diverse Molecular Stacking Enable High-Performance Organic Solar Cells with V_{OC} Over 1.1 V. *Sci. China Chem.* **67**, 3140–3152 (2024).

33. Zhang, L. *et al.* Regulating Pre-Aggregation in Non-Halogenated Solvent to Enhance the Efficiency of Organic Solar Cells. *Appl. Phys. Lett.* **124**, 013902 (2024).

34. Zhang, L. *et al.* Regulation of Crystallinity and Vertical Phase Separation Enables High-Efficiency Thick Organic Solar Cells. *Adv. Funct. Mater.* **32**, 2202103 (2022).

35. PanFeng, G., LiYong, W., HaiYan, F. & Yuan, D. Synthesis, Characterizations and Photovoltaic Applications of a Thickness-Insensitive Benzodifuran Based Copolymer. *Eur. Polym. J.* **172**, 111189 (2022).

36. Xie, X. *et al.* Thickness Insensitive Organic Solar Cells with High Figure-of-Merit-X Enabled by Simultaneous D/A Interpenetration and Stratification. *Adv. Energy Mater.* **14**, 2401355 (2024).

37. Cheng, X. *et al.* “Twisted” Small Molecule Donors with Enhanced Intermolecular Interactions in the Condensed Phase Towards Efficient and Thick-Film All-Small-Molecule Organic Solar Cells. *J. Mater. Chem. A* **11**, 13984–13993 (2023).

38. Cai, Y. *et al.* Vertically Optimized Phase Separation with Improved Exciton Diffusion Enables Efficient Organic Solar Cells with Thick Active Layers. *Nat. Commun.* **13**, 2369 (2022).
39. Zhang, J. *et al.* π-Extended Conjugated Polymer Acceptor Containing Thienylene–Vinylene–Thienylene Unit for High-Performance Thick-Film All-Polymer Solar Cells with Superior Long-Term Stability. *Adv. Energy Mater.* **11**, 2102559 (2021).
40. Wu, X. *et al.* 19.36% Efficiency Organic Solar Cells Based on Low-Cost Terpolymer Donors with Simple Molecular Structures. *Adv. Funct. Mater.* **34**, 2405168 (2024).
41. Su, Z. *et al.* A 3,3′-Difluoro-2,2′-Bithiophene Based Donor Polymer Realizing High Efficiency (>17%) Single Junction Binary Organic Solar Cells. *Small* **20**, 2310028 (2024).
42. Zhang, S. *et al.* A Large Area Organic Solar Module with Non-Halogen Solvent Treatment, High Efficiency, and Decent Stability. *Sol. RRL* **7**, 2300029 (2023).
43. Zhang, L., Zhang, Z., Liang, H., Guo, X. & Zhang, M. A Non-Halogenated Polymer Donor Based on Imide Unit for Organic Solar Cells with Efficiency Nearly 16%. *Chinese J. Chem.* **41**, 2095–2102 (2023).
44. Chen, S. *et al.* A Nonfullerene Semitransparent Tandem Organic Solar Cell with 10.5% Power Conversion Efficiency. *ACS Energy Lett.* **8**, 1800529 (2018).
45. Zhu, J. *et al.* A-D-A Type Nonfullerene Acceptors Synthesized by Core

Segmentation and Isomerization for Realizing Organic Solar Cells with Low Nonradiative Energy Loss. *Small* **20**, 2305529 (2024).

46. Zhu, L. *et al.* Achieving 20.8% Organic Solar Cells via Additive-Assisted Layer-by-Layer Fabrication with Bulk *p-i-n* Structure and Improved Optical Management. *Joule* **8**, 3153–3168 (2024).
47. Zhang, L. *et al.* Achieving Balanced Crystallinity of Donor and Acceptor by Combining Blade-Coating and Ternary Strategies in Organic Solar Cells. *Adv. Mater.* **30**, 1805041 (2018).
48. Ma, S. *et al.* Biselenophene Imide: Enabling Polymer Acceptor with High Electron Mobility for High-Performance All-Polymer Solar Cells. *Angew. Chem. Int. Ed.* **62**, e202308306 (2023).
49. Wei, N. *et al.* Constructing Multiscale Fibrous Morphology to Achieve 20% Efficiency Organic Solar Cells by Mixing High and Low Molecular Weight D18. *Adv. Mater.* **36**, 2408934 (2024).
50. Pradhan, R. *et al.* Correlation of Functional Coumarin Dye Structure with Molecular Packing and Organic Solar Cells Performance. *Sol. RRL* **7**, 2300487 (2023).
51. Wang, J. *et al.* Cyanoesterthiophene Based Low-Cost Polymer Donors for High Efficiency Organic Solar Cells. *Adv. Funct. Mater.* **34**, 2313850 (2024).
52. Li, C. *et al.* Cyclization of Inner Linear Alkyl Chains in Fused-Ring Electron

Acceptors Toward Efficient Organic Solar Cells. *Sol. RRL* **7**, 2300067 (2023).

53. Lee, J.-W. *et al.* Design of Star-Shaped Trimer Acceptors for High-Performance (Efficiency > 19%), Photostable, and Mechanically Robust Organic Solar Cells. *Adv. Energy Mater.* **14**, 2303872 (2024).
54. Lee, J.-W. *et al.* Efficient and Photostable Organic Solar Cells Achieved by Alloyed Dimer Acceptors with Tailored Linker Structures. *Adv. Funct. Mater.* **34**, 2404569 (2024).
55. Lei, H. *et al.* Electron Transporting Polymeric Materials with Partial Quaternization for High-Performance Organic Solar Cells. *Macromol. Rapid Commun.* **45**, 2400479 (2024).
56. Cui, Y. *et al.* Eliminating the Imbalanced Mobility Bottlenecks via Reshaping Internal Potential Distribution in Organic Photovoltaics. *Adv. Sci.* **10**, 2302880 (2023).
57. Cheng, Y. *et al.* Enabling High-Efficiency and Stable Binary Organic Solar Cells by Solid Additive-Assisted Morphology Modulation. *Adv. Funct. Mater.* **n/a**, 2415468.
58. Zhang, F. *et al.* Enhanced and Balanced Carrier Mobility Via n-Type SnS Dopant Enables High-Performance Non-Fullerene Organic Solar Cells. *Adv. Funct. Mater.* **34**, 2406066 (2024).
59. Wang, H., Wang, X., Fan, P., Yang, X. & Yu, J. Enhanced Power Conversion

Efficiency of P3HT : $PC_{71}BM$ Bulk Heterojunction Polymer Solar Cells by Doping a High-Mobility Small Organic Molecule. *Int. J. Photoenergy* **2015**, 982064 (2015).

60. Wu, X. *et al.* Enhancing Efficiency and Stability in Organic Photovoltaics through Miscibility of High-Tg Insulating Material with Y-Series Nonfullerene Acceptors. *Sol. RRL* **8**, 2300812 (2024).
61. Bi, P. *et al.* Enhancing Photon Utilization Efficiency for High-Performance Organic Photovoltaic Cells via Regulating Phase-Transition Kinetics. *Adv. Mater.* **35**, 2210865 (2023).
62. Liu, B. *et al.* Enhancing Photovoltaic Performance of Nonfused-Ring Electron Acceptors via Asymmetric End-Group Engineering and Noncovalently Conformational Locks. *Chin. J. Chem.* **42**, 485–490 (2024).
63. Liu, D. *et al.* Extended Conjugation Length of Nonfullerene Acceptors with Improved Planarity via Noncovalent Interactions for High-Performance Organic Solar Cells. *Adv. Energy Mater.* **8**, 1801618 (2018).
64. Shi, J. *et al.* Facile Side Chains Optimization of Y-series Acceptor Enables High Performance Binary Non-halogenated Solvent-Processed Organic Solar Cells with Excellent Fill Factor of 79%. *Sol. RRL* **7**, 2300206 (2023).
65. Deng, J. *et al.* Ferroelectric Polymer Drives Performance Enhancement of Non-fullerene Organic Solar Cells. *Angew. Chem. Int. Ed.* **61**, e202202177 (2022).
66. Fang, H. *et al.* Fullerene-Hybridized Fused-Ring Electron Acceptor with High

Dielectric Constant and Isotropic Charge Transport for Organic Solar Cells. *Angew. Chem. Int. Ed.* **n/a**, e202417951.

67. Zhu, T. *et al.* Functionality of Non-Fullerene Electron Acceptors in Ternary Organic Solar Cells. *Sol. RRL* **3**, 1900322 (2019).
68. Yuan, J. *et al.* Fused Benzothiadiazole: A Building Block for n-Type Organic Acceptor to Achieve High-Performance Organic Solar Cells. *Adv. Mater.* **31**, 1807577 (2019).
69. Zhuo, H. *et al.* Giant Molecule Acceptor Enables Highly Efficient Organic Solar Cells Processed Using Non-halogenated Solvent. *Angew. Chem. Int. Ed.* **62**, e202303551 (2023).
70. Liu, L. *et al.* Graphdiyne Derivative as Multifunctional Solid Additive in Binary Organic Solar Cells with 17.3% Efficiency and High Reproductivity. *Adv. Mater.* **32**, 1907604 (2020).
71. Li, D. *et al.* Halogenated Nonfused Ring Electron Acceptor for Organic Solar Cells with a Record Efficiency of over 17%. *Adv. Mater.* **36**, 2310362 (2024).
72. Chen, Q. *et al.* Hierarchical Solid-Additive Strategy for Achieving Layer-by-Layer Organic Solar Cells with Over 19 % Efficiency. *Angew. Chem. Int. Ed.* **63**, e202405949 (2024).
73. Tan, J. *et al.* High-Performance Organic Solar Cells by Adding Two-Dimensional GeSe. *Adv. Funct. Mater.* **32**, 2209094 (2022).

74. Chen, J. *et al.* Highly Efficient and Stable Organic Solar Cells Enabled by a Commercialized Simple Thieno[3,2-b]thiophene Additive. *Small Methods* **8**, 2400172 (2024).

75. Chen, C. *et al.* Molecular Interaction Induced Dual Fibrils Towards Organic Solar Cells with Certified Efficiency Over 20%. *Nat. Commun.* **15**, 6865 (2024).

76. Jiang, Y. *et al.* Non-Fullerene Acceptor with Asymmetric Structure and Phenyl-Substituted Alkyl Side Chain for 20.2% Efficiency Organic Solar Cells. *Nat. Energy* **9**, 975–986 (2024).

77. Yi, F. *et al.* Non-Fully Conjugated Dimerized Giant Acceptors with Different Alkyl-Linked Sites for Stable and 19.13 % Efficiency Organic Solar Cells. *Angew. Chem. Int. Ed.* **63**, e202319295 (2024).

78. Privado, M. *et al.* Noncovalent Conformational Locks Enabling Efficient Nonfullerene Acceptors. *Sol. RRL* **6**, 2100768 (2022).

79. Gao, K. *et al.* Over 12% Efficiency Nonfullerene All-Small-Molecule Organic Solar Cells with Sequentially Evolved Multilength Scale Morphologies. *Adv. Mater.* **31**, 1807842 (2019).

80. Chen, X. *et al.* Polymer Donor with a Simple Skeleton and Minor Siloxane Decoration Enables 19% Efficiency of Organic Solar Cells. *Adv. Mater.* **36**, 2313074 (2024).

81. Han, Z. *et al.* Precisely Manipulating Molecular Packing via Tuning Alkyl Side-

Chain Topology Enabling High-Performance Nonfused-Ring Electron Acceptors. *Angew. Chem. Int. Ed.* **63**, e202318143 (2024).

82. Sun, C. *et al.* Regiospecific Incorporation of Acetylene Linker in High-Electron Mobility Dimerized Acceptors for Organic Solar Cells with High Efficiency (18.8%) and Long 1-Sun Lifetime (> 5000 h). *Adv. Energy Mater.* **13**, 2301283 (2023).
83. Luo, X. *et al.* Self-Doped Conjugated Polymers with Electron-Deficient Quinone Units for Enhanced Electron Transport in Highly Efficient Organic Solar Cells. *FlexMat* **1**, 105–115 (2024).
84. Wang, X. *et al.* Simple Nonfused Ring Electron Acceptors with 3D Network Packing Structure Boosting the Efficiency of Organic Solar Cells to 15.44%. *Adv. Energy Mater.* **11**, 2102591 (2021).
85. Liu, L. *et al.* Stereoisomeric Non-Fullerene Acceptors-Based Organic Solar Cells. *Small* **20**, 2305638 (2024).
86. Shi, K. *et al.* Synergistic Effects of Solid and Solvent Additives on Film Morphology Enable Binary Organic Solar Cells with Efficiency of Over 19%. *Adv. Funct. Mater.* **35**, 2411787 (2025).
87. Zhou, L. *et al.* Tailoring the Position of Ester Group on N-Alkyl Chains of Benzotriazole-based Small Molecule Acceptors for High-Performance Organic Solar Cells. *Angew. Chem. Int. Ed.* **63**, e202319635 (2024).
88. Liao, C. *et al.* Tetrahydrofuran Processable Organic Solar Cells with 19.45%

Efficiency Realized by Introducing High Molecular Dipole Unit into the Terpolymer. *Adv. Mater.* **36**, 2411071 (2024).

89. Cheng, Y. *et al.* Three-in-One Strategy Enables Single-Component Organic Solar Cells with Record Efficiency and High Stability. *Adv. Mater.* **36**, 2312938 (2024).
90. Ren, J. *et al.* TVT-Based New Building Block with Enhanced π-Electron Delocalization for Efficient Non-Fused Photovoltaic Acceptor. *Small Methods* **n/a**, 2401511.
91. Zhang, Y. *et al.* Unraveling the Role of Non-Fullerene Acceptor with High Dielectric Constant in Organic Solar Cells. *Small* **19**, 2302314 (2023).
92. Zhu, C. *et al.* Vertical Phase Regulation with 1,3,5-Tribromobenzene Leads to 18.5% Efficiency Binary Organic Solar Cells. *Adv. Sci.* **10**, 2303150 (2023).
93. Sun, Y. *et al.* π-Extended Nonfullerene Acceptor for Compressed Molecular Packing in Organic Solar Cells To Achieve over 20% Efficiency. *J. Am. Chem. Soc.* **146**, 12011–12019 (2024).
