# Supplementary material for: Critical length screening enables 19% efficiency in thick-film organic solar cells
Source: Nat Commun. 2025 Nov 7;16:9840. doi: 10.1038/s41467-025-64808-x (PMC12594902; doi:10.1038/s41467-025-64808-x)
Supplement: Supplementary file 2 — Reporting Summary [file 41467_2025_64808_MOESM2_ESM.pdf]

nature portfolio

Corresponding author(s): Hang Yin, NCOMMS-25-39270B

Solar Cells Reporting Summary

Nature Portfolio wishes to improve the reproducibility of the work that we publish. This form is intended for publication with all accepted papers reporting the characterization of photovoltaic devices and provides structure for consistency and transparency in reporting. Some list items might not apply to an individual manuscript, but all fields must be completed for clarity.

For further information on Nature Research policies, including our data availability policy, see Authors & Referees.

Experimental design

Please check the following details are reported in the manuscript, and provide a brief description or explanation where applicable.

1. Dimensions

Item	Yes/No	Response
Area of the tested solar cells	☒ Yes ☐ No	The area of devices manufactured by us is 0.052 cm^2. / *Explain why this information is not reported/not relevant.*
Method used to determine the device area	☒ Yes ☐ No	Photovoltaic cells were fabricated on one substrate and the active area of each device was 0.052 cm^2 defined by a shadow mask. / *Explain why this information is not reported/not relevant.*

2. Current-voltage characterization

Item	Yes/No	Response
Current density-voltage (J-V) plots in both forward and backward direction	☐ Yes ☒ No	Since the OSC performance does not depend on the scanning direction, we only measure the device in the forward direction.
Voltage scan conditions	☒ Yes ☐ No	We used a step voltage of 0.02 V and a dwell time of 0.02 s for each voltage. / *Explain why this information is not reported/not relevant.*
Test environment	☒ Yes ☐ No	The J-V testing was accomplished in a glove box in a nitrogen atmosphere with an ambient temperature of 25 °C and 0% humidity. / *Explain why this information is not reported/not relevant.*
Protocol for preconditioning of the device before its characterization	☐ Yes ☒ No	*Provide a description of the protocol.* / No preconditioning protocol was used before testing.
Stability of the J-V characteristic	☐ Yes ☒ No	*Provide a description of the method used. The stability of the J-V characteristic can be verified with time evolution of the maximum power point or with the photocurrent at maximum power point; see ref. 5 for details.* / We didn't provide the information since the proposed strategy theoretically has no influence on stability of J-V characteristic.

3. Hysteresis or any other unusual behaviour

Item	Yes/No	Response
Description of the unusual behaviour observed during the characterization	☐ Yes ☒ No	*Provide a description of hysteresis or any other unusual behaviour observed during the characterization.* / No unusual behaviour was observed during the characterization.
Related experimental data	☐ Yes ☒ No	*Provide a description of the related experimental data.* / No related experimental data need to be attached.

4. Efficiency

Item	Yes/No	Response
External quantum efficiency (EQE) or incident photons to current efficiency (IPCE)	☒ Yes ☐ No	EQE spectra of key representative devices were shown in Figure 5 and Figure S7. / *Explain why this information is not reported/not relevant.*
A comparison between the integrated response under the standard reference spectrum and the response measure under the simulator	☒ Yes ☐ No	The integrated current density from EQE spectra is within 4% of the JV measured Jsc. / *Explain why this information is not reported/not relevant.*

For tandem solar cells, the bias illumination and bias voltage used for each subcell

- [ ] Yes
- [x] No

Provide a description of the measurement conditions.

We did not fabricate tandem devices.

5. Calibration

Light source and reference cell or sensor used for the characterization

- [x] Yes
- [ ] No

The J-V curves of all devices were measured under an illumination of AM 1.5G (100 mW/cm2) using a Keithley 2400 source meter generating from LSS-55 solar simulator (50*50 mm spot size) of LightSky Technology CO., LTD.

Explain why this information is not reported/not relevant.

Confirmation that the reference cell was calibrated and certified

- [ ] Yes
- [x] No

Identify the independent certification laboratory.

The reference cell was calibrated but has not been certified by an independent certification laboratory.

Calculation of spectral mismatch between the reference cell and the devices under test

- [x] Yes
- [ ] No

Spectral mismatch between reference cell and testing cells was less than 5%.

Explain why this information is not reported/not relevant.

6. Mask/aperture

Size of the mask/aperture used during testing

- [x] Yes
- [ ] No

During the test, an aperture with an area of 0.041 cm^2 is used to calibrate the device area.

Explain why this information is not reported/not relevant.

Variation of the measured short-circuit current density with the mask/aperture area

- [ ] Yes
- [x] No

Report the difference in the short-circuit current density values measured with the mask and aperture area.

The variation of short-circuit current density with aperture area was not considered in this work.

7. Performance certification

Identity of the independent certification laboratory that confirmed the photovoltaic performance

- [ ] Yes
- [x] No

Identify the independent certification laboratory.

Explain why this information is not reported/not relevant.

A copy of any certificate(s)

- [ ] Yes
- [x] No

Certificate copies should be provided in the Supplementary information. Please state the supplementary item number.

Explain why this information is not reported/not relevant.

8. Statistics

Number of solar cells tested

- [x] Yes
- [ ] No

The averaged values are calculated from 10 independent cells from different batches.

Explain why this information is not reported/not relevant.

Statistical analysis of the device performance

- [x] Yes
- [ ] No

This information can be found in manuscript.

Explain why this information is not reported/not relevant.

9. Long-term stability analysis

Type of analysis, bias conditions and environmental conditions

- [ ] Yes
- [x] No

Provide a description of the type of analysis, bias conditions and environmental conditions (e.g. illumination type, temperature, atmosphere humidity, encapsulation method, preconditioning temperature, bias) for each long-term stability analysis carried out; see ref. 7 and 8 for details.

As we mainly propose a new method for screening acceptor materials in high-efficiency thick-film organic solar cells, we have not done stability measurements on our devices.
